# Synthesis, Characterization, and Multifunctional Bioactivity of ZnO/CuO@Amoxicillin Nanocomposites: A Synergistic Nano‐Platform With Potent Antioxidant, Anti‐Inflammatory, Antidiabetic, and Anti‐Cholinesterase Activities

**DOI:** 10.1002/cbdv.71658

**Published:** 2026-09-03

**Authors:** Hocine Sadam Nesrat, Abdelatif Aouadi, Ibtissam Laib, Salah Eddine Laouini, Abderrhmane Bouafia, Djamila Hamada Saoud, Halima Zidane, Hafidha Terea, Mahmood M. S. Abdullah, Tomasz Trzepieciński, Dalila Saidane‐Mosbahi

**Affiliations:** ^1^ Laboratory of Analysis, Treatment and Recovery of Environmental Pollutants and Products, Faculty of Pharmacy University of Monastir Monastir Tunisia; ^2^ Laboratory of Biotechnology Biomaterials and Condensed Matter (LBBCM), Faculty of Technology University of El Oued El Oued Algeria; ^3^ Process Engineering Laboratory, Applied Sciences Faculty Kasdi Merbah University Ouargla Algeria; ^4^ Faculty of Biology, Department of Cellular and Molecular Biology University of El Oued El Oued Algeria; ^5^ Laboratory of Biodiversity and Application of Biotechnology in the Agricultural Field, Faculty of Natural and Life Sciences University of El Oued El Oued Algeria; ^6^ Department of Process Engineering and Petrochemistry, Faculty of Technology University of El Oued El Oued Algeria; ^7^ Laboratory Valorization and Technology of Saharan Resources (VTRS) University of El Oued El Oued Algeria; ^8^ Department of Chemistry College of Science King Saud University Riyadh Saudi Arabia; ^9^ Department of Manufacturing Processes and Production Engineering Rzeszów University of Technology Rzeszów Poland

**Keywords:** amoxicillin functionalization, antidiabetic, anti‐inflammatory, antioxidant activity, neuroprotective, ZnO/CuO nanocomposite

## Abstract

A ZnO/CuO@amoxicillin nanocomposite was synthesized via a sol–gel route and characterized as a biphasic wurtzite ZnO–monoclinic CuO system with crystallite sizes of about 10–12 nm and an optical band gap of 3.30–3.36 eV, indicating defect‐rich interfaces favorable for redox activity. In antidiabetic assays, the nanocomposite showed strong inhibitory effects on alpha‐amylase (IC_50_ = 21.57 ± 1.68 mg/mL) and alpha‐glucosidase (IC_50_ = 13.95 ± 0.76 mg/mL), with activities comparable to acarbose, while anti‐inflammatory tests yielded low IC_50_ values of 0.22 ± 0.03 mg/mL for egg albumin denaturation and 0.34 ± 0.07 mg/mL for human serum albumin, approaching the performance of diclofenac. Antioxidant performance was outstanding, with total antioxidant capacity of 298.27 ± 4.70 mg ascorbic acid equivalents per gram and ferric reducing antioxidant power of 311.67 ± 2.53 mM Fe^2^^+^/g, both higher than ascorbic acid, alongside efficient hydrogen peroxide scavenging (IC_50_ = 0.16 ± 0.04 mg/mL) and protection against H_2_O_2_/FeCl_3_‐induced hemolysis at an IC_50_ of 0.007 ± 0.00 mg/mL; together with moderate but significant acetylcholinesterase inhibition (IC_50_ = 33.16 ± 3.02 mg/mL vs. 21.80 ± 2.74 mg/mL for donepezil), these results support ZnO/CuO@amoxicillin as a multifunctional nano‐platform for managing oxidative stress, inflammation, and metabolic disorders.

## Graphical abstract

ZnO/CuO@amoxicillin nanocomposite, synthesized via sol–gel route, demonstrates exceptional multifunctional performance. It strongly inhibits α‐amylase and α‐glucosidase, effectively suppresses inflammation, and exhibits superior antioxidant activity surpassing ascorbic acid. With potent protection against oxidative hemolysis and moderate acetylcholinesterase inhibition, this nano‐platform offers a promising integrated approach for managing diabetes, oxidative stress, and inflammatory disorders.

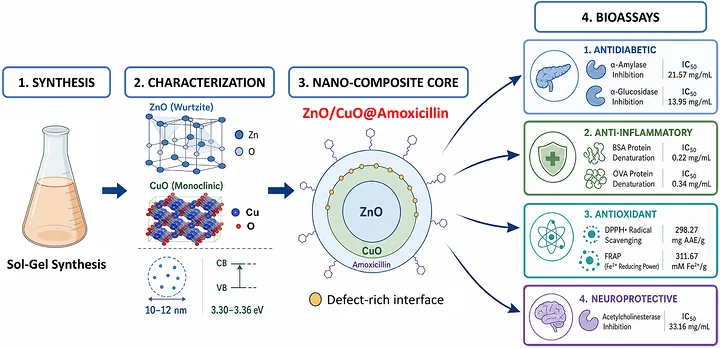

## 1 Introduction

In recent years, metal oxide nanoparticles have gained prominence in biomedical research due to their stability, availability, and diverse biological effects. Copper oxide (CuO) and zinc oxide (ZnO) nanoparticles are among the most studied, owing to their nanoscale reactivity and multifaceted interactions with microbial cells, including reactive oxygen species generation, metal ion release, and surface interactions, rendering them valuable for antioxidant, antibacterial, and anti‐inflammatory applications. Studies indicate that integrating CuO and ZnO into a single nanocomposite significantly enhances their efficacy through synergistic complementarity [1, 2, 3, 4]. Additionally, loading antibiotics onto metal oxide nanoparticles represents a promising strategy to improve drug stability and targeted delivery. Amoxicillin, a widely used β‐lactam antibiotic, exhibits superior performance when conjugated to nanostructured metal oxide carriers [5, 6]. This approach enables the development of multifunctional systems capable of simultaneous drug delivery and biological activity contribution. It should be emphasized that the expected antioxidant, anti‐inflammatory, antidiabetic, and AChE inhibitory activities in this study are not attributed to amoxicillin alone, but rather to the synergistic contribution of the ZnO/CuO nanocomposite and its functionalization with amoxicillin. Previous reports have indicated that β‐lactam antibiotics may indirectly modulate oxidative stress and inflammatory responses [7], while metal oxide nanostructures provide intrinsic redox and enzyme‐modulatory properties [8, 9]. Therefore, the hybrid ZnO/CuO@amoxicillin system is anticipated to exhibit multifunctional bioactivities through combined nano–bio interactions.

The escalating antimicrobial resistance crisis, driven by widespread antibiotic exposure in healthcare, agriculture, and the environment, has globally selected for resistant bacterial strains, reducing the clinical efficacy of many antibiotics [10]. Resistance to β‐lactam antibiotics such as amoxicillin primarily arises from β‐lactamase‐mediated enzymatic degradation, decreased membrane permeability, and target site modifications, which impair drug access and binding [11, 12]. Furthermore, biofilm formation imposes physical and physiological barriers that limit antibiotic penetration and promote persister cells, contributing to chronic and recalcitrant infections [13]. Consequently, nanoparticle‐based strategies have gained prominence, with metal oxide nanoparticles serving as intrinsic antimicrobials and carriers that enhance antibiotic stability, delivery, and synergistic effects to potentially overcome resistance mechanisms [1, 14].

Combining CuO and ZnO into a single nanocomposite provides distinct advantages over individual nanoparticles, owing to their complementary properties: CuO imparts potent antibacterial and catalytic activity, while ZnO enhances stability, biocompatibility, and antioxidant effects [15, 16, 17]. This synergy amplifies overall biological efficacy and improves physicochemical stability of the nanostructure. Loading amoxicillin onto such nanocomposites further elevates their multifunctional therapeutic potential by protecting the drug from premature degradation, enhancing solubility, and promoting efficient bacterial cell interactions [18, 19]. Additionally, the metal oxides facilitate biofilm penetration and may circumvent certain resistance mechanisms, thereby augmenting antimicrobial and anti‐inflammatory activity [20, 21]. Consequently, CuO/ZnO@amoxicillin nanocomposites emerge as promising candidates for tackling complex biomedical challenges through integration of inherent oxide bioactivity with targeted antibiotic delivery.

The synthesis method critically influences the properties and performance of nanocomposites. Among various approaches, the sol–gel technique has emerged as a preferred method for fabricating metal oxide nanoparticles, offering precise control over particle size, morphology, uniformity, and purity [22, 23]. This process entails the transformation of a colloidal sol into a gel network via hydrolysis and condensation reactions, enabling tailored structural and surface properties. For CuO/ZnO nanocomposites, sol–gel synthesis promotes homogeneous oxide distribution and yields high surface area, facilitating efficient drug loading and biological target interactions [24, 25]. Moreover, the mild processing conditions preserve the integrity of sensitive therapeutics such as amoxicillin during incorporation, thereby improving drug stability and bioavailability [26]. Consequently, the sol–gel method substantially enhances the development of multifunctional CuO/ZnO@amoxicillin systems with optimized biological efficacy.

Despite promising individual bioactivities of CuO and ZnO nanoparticles, few studies have explored their combined nanocomposite loaded with antibiotics for multifunctional applications. Previous work mainly addressed monometallic oxide synthesis or limited bioactivities (e.g., antibacterial/antioxidant), neglecting broader therapeutic potentials [27, 28, 29]. Systematic evaluations of amoxicillin‐loaded CuO/ZnO nanocomposites for diverse activities such as AChE inhibition, anti‐inflammatory effects, hemolysis protection, β‐carotene bleaching inhibition, anti‐diabetic potential, and multiple antioxidant assays are particularly scarce.

This study thus synthesizes an amoxicillin‐loaded CuO/ZnO nanocomposite via sol–gel, characterizes it using XRD, SEM, FTIR, and UV–vis, and assesses its multifaceted biological activities to establish CuO/ZnO@amoxicillin as a versatile nanoplatform against antimicrobial resistance and other biomedical challenges.

This study reports the first successful synthesis and characterization of a dual‐phase ZnO/CuO nanocomposite functionalized with amoxicillin (ZnO/CuO@amoxicillin) via a cost‐effective sol–gel method, designed to address the urgent need for multifunctional therapeutic agents. Unlike previous studies that focused primarily on single‐metal oxides or non‐functionalized heterostructures, this work introduces a novel organic–inorganic hybrid where the formation of a p–n heterojunction between wurtzite ZnO and monoclinic CuO is synergistically coupled with the antibiotic amoxicillin. The novelty lies in the significant modulation of the electronic band structure, evidenced by a tunable optical band gap of 3.30–3.36 eV and a high Urbach energy of 0.346 eV. This specific electronic configuration creates a defect‐rich interface that facilitates superior electron–hole separation and redox activity compared to individual CuO or ZnO nanoparticles. Furthermore, this research is unique in demonstrating that such structural engineering translates directly into broad‐spectrum biological efficacy, achieving simultaneous antidiabetic, anti‐inflammatory, and antioxidant activities at levels comparable to standard drugs like acarbose and diclofenac. By integrating these diverse bioactivities into a single, stable nanoplatform with verified low‐dose cytoprotection (hemolysis IC_50_ of 0.007 mg/mL), this work overcomes the limitations of conventional monofunctional therapies and offers a pioneering strategy for treating complex pathologies involving oxidative stress, metabolic dysregulation, and inflammation.

## 2 Materials and Methods

### 2.1 Chemicals and Reagents

All chemicals and biological reagents were of analytical grade and used without further purification. The synthesis of the ZnO/CuO nanocomposite and drug loading procedures employed the following: copper(II) nitrate trihydrate (Cu(NO_3_)_2_·3H_2_O, 98%), zinc acetate dihydrate (Zn(CH_3_COO)_2_·2H_2_O, 99%), sodium hydroxide (NaOH, 99.8%), absolute ethanol (C_2_H_5_OH, 99.9%), citric acid, ethylene glycol, deionized water, and amoxicillin.

For antioxidant evaluation, the following reagents were used: a methanolic solution of 1,1‐diphenyl‐2‐picrylhydrazyl (DPPH•, 0.1 mM); ascorbic acid as a reference standard; sulfuric acid (H_2_SO_4_, 6 M); sodium phosphate (NaH_2_PO_4_, 28 mM); ammonium molybdate ((NH_4_)_2_MoO_4_, 4 mM) for the phosphomolybdenum assay; phosphate buffer (0.2 M, pH 6.6); potassium ferricyanide (K_3_[Fe(CN)_6_], 1%); trichloroacetic acid (TCA, 10%); ferric chloride (FeCl_3_, 0.1%); ABTS•^+^ radical cation solution (2 mM), prepared by oxidation with potassium persulfate (K_2_S_2_O_8_, 70 mM); β‐carotene; linoleic acid; Tween 40; chloroform (CHCl_3_); riboflavin (50 mM); phenazine methosulfate (PMS, 20 mM); nitro blue tetrazolium (NBT, 5 mM) for superoxide anion scavenging; hydrogen peroxide (H_2_O_2_, 200 mM); 2‐deoxyribose (2.8 mM); ethylenediaminetetraacetic acid (EDTA, 100 mM); thiobarbituric acid (TBA, 1%); and phosphate‐buffered saline (PBS, pH 7.4) prepared from NaCl, Na_2_HPO_4_, and KCl.

Hemolysis assays were performed using human blood samples (O+ type), with ferric chloride (FeCl_3_, 80 mM) and hydrogen peroxide (H_2_O_2_, 30 mM) as inducing agents.

Anti‐inflammatory activity was assessed using the egg albumin denaturation assay and a human serum albumin (HSA, 5%) model in PBS, with diclofenac sodium as the standard drug.

For antidiabetic assays, the following were utilized: α‐glucosidase enzyme (1 U/mL) with p‐nitrophenyl‐α‐d‐glucopyranoside (p‐NPG, 5 mM) as substrate; and α‐amylase enzyme (2 U/mL) with soluble potato starch (1%) as substrate, using the dinitro‐salicylic acid (DNS) method for reducing sugar determination.

Acetylcholinesterase (AChE) inhibitory activity was evaluated using an acetylcholinesterase enzyme solution in phosphate buffer (0.1 M Na_2_HPO_4_, pH 8), with 5,5'‐dithiobis(2‐nitrobenzoic acid) (DTNB) and acetylthiocholine iodide (ACTHI) as substrates.

### 2.2 Synthesis of ZnO/CuO Nanocomposite and Drug Loading

The ZnO/CuO nanocomposite was synthesized via a sol–gel method. Briefly, equimolar solutions of Cu(NO_3_)_2_·3H_2_O and Zn(CH_3_COO)_2_·2H_2_O were prepared in an ethanol–water mixture (1:1 v/v), with citric acid and ethylene glycol added as complexing agents. The combined solution was stirred vigorously, and the pH was adjusted to ∼9 using NH_4_OH. The resulting sol was aged for 24 h at room temperature. After solvent exchange with ethanol, gelation was performed at 60°C for 12 h, followed by calcination in a stepwise manner: 200°C for 2 h, 350°C for 1 h, and finally 450°C for 3 h under oxygen flow. This yielded a phase‐pure nanocomposite of wurtzite ZnO and monoclinic CuO [30, 31].

For drug loading, 100 mg of the nanocomposite was dispersed in 50 mL of amoxicillin solution (1 mg/mL in phosphate buffer, pH 7.4). The mixture was sonicated and stirred for 12 h at 25°C. The solid was then collected by centrifugation (8000 rpm, 10 min), washed repeatedly with buffer, and vacuum‐dried at 40°C–50°C [32, 33]. Loading efficiency was determined by UV–vis analysis of the supernatant, and successful amoxicillin adsorption was confirmed by FTIR. SEM imaging revealed a porous morphology conducive to high drug loading capacity.

### 2.3 Characterization

For SEM analysis, the nanocomposite powders were sputter‐coated with a thin layer of gold to improve conductivity and prevent charging effects during imaging. Microstructural observations were carried out using a JEOL JSM‐6510LV scanning electron microscope operated at 20 kV. For XRD measurements, samples were analyzed using a Bruker D8 Advance diffractometer equipped with Cu Kα radiation (*λ* = 1.5406 Å). Data were collected over a 2*θ* range of 20°–80° with a step size of 0.02° to determine phase composition and crystallite size. For FTIR spectroscopy, samples were finely ground, mixed with KBr, and pressed into pellets. Spectra were recorded using a PerkinElmer Spectrum Two instrument in the range of 400–4000 cm−^1^ to identify functional groups and confirm drug incorporation. These detailed procedures ensure reproducibility and scientific rigor of the findings.

### 2.4 Antioxidant Capacity

#### 2.4.1 DPPH Radical Scavenging Assay

The DPPH radical scavenging activity of the ZnO/CuO@amoxicillin nanocomposite was assessed according to the method of Brand‐Williams et al. [34]. Briefly, 1 mL of the nanocomposite suspension (at varying concentrations) was mixed with 1 mL of a methanolic DPPH solution (0.1 mM). The mixture was incubated in the dark at room temperature for 15 min. The absorbance was then measured at 517 nm using a UV–vis spectrophotometer. Ascorbic acid (0.005–0.5 mg/mL) served as the reference standard.

The percentage inhibition was calculated using Equation (1):

(1) $$\%Inhibition=\left[\frac{\left(A_{control}-A_{sample}\right)}{A_{control}}\right]\;\times 100$$

where $A_{control}$ and $A_{sample}$ represent the absorbance of the control and sample, respectively. The IC_50_ value (concentration required for 50% inhibition) was determined from a linear regression plot of inhibition percentage versus concentration.

#### 2.4.2 Total Antioxidant Capacity (TAC)

The total antioxidant capacity was determined using the phosphomolybdenum method [35]. A 1 mL sample was combined with 2 mL of reagent solution (6 M H_2_SO_4_, 28 mM NaH_2_PO_4_, 4 mM (NH_4_)_2_MoO_4_) and incubated at 95°C for 90 min. After cooling, the absorbance was measured at 695 nm. Results are expressed as mg ascorbic acid equivalents per gram of nanocomposite.

#### 2.4.3 Ferric Reducing Antioxidant Power (FRAP)

The reducing power was assessed as described [36]. Briefly, 250 µL of sample was mixed with 625 µL of phosphate buffer (0.2 M, pH 6.6) and 625 µL of 1% K_3_[Fe(CN)_6_], then incubated at 50°C for 20 min. After adding 525 µL of 10% trichloroacetic acid and centrifugation, 125 µL of 0.1% FeCl_3_ was added to the supernatant. Absorbance was measured at 700 nm, with ascorbic acid as a standard. Higher absorbance indicates greater reducing power [37].

#### 2.4.4 ABTS Radical Scavenging Assay

ABTS•^+^ scavenging activity was evaluated following a reported method [38]. The ABTS•^+^ radical cation was generated by reacting 2 mM ABTS in PBS (pH 7.4) with 70 mM K_2_S_2_O_8_ for 1 h in the dark. The solution was diluted to an absorbance of 0.700 ± 0.020 at 734 nm. For the assay, 50 µL of sample was added to 3 mL of this working solution, and absorbance was measured at 734 nm [39]. The inhibition percentage was calculated using Equation (2):

(2) $$I\%\;=\frac{\left(A_{\mathrm{control}}\;-\;A_{\mathrm{sample}}\right)}{A_{\mathrm{control}}}\times \;100$$

where $\;A_{\mathrm{sample}}$ is the absorbance of the sample; $A_{\mathrm{control}}$ is the absorbance of the control that is, lack of inhibitor.

##### 2.4.4.1 β‐Carotene/Linoleic Acid Bleaching Assay

The antioxidant activity of the nanocomposite was further evaluated using the β‐carotene‐linoleic acid bleaching assay according to a modified method [40]. Briefly, a β‐carotene emulsion was prepared by dissolving 0.2 mg β‐carotene, 20 mg linoleic acid, and 200 mg Tween 40 in 0.5 mL chloroform. The solvent was evaporated under reduced pressure, and the residue was vigorously emulsified in 50 mL of oxygenated distilled water.

For the assay, 4 mL of this emulsion was mixed with 0.2 mL of the sample solution. The mixture was incubated at 50°C in a water bath, and the absorbance at 470 nm was measured immediately (*t* = 0) and at 15‐minute intervals for 180 min. A control without β‐carotene was included. The antioxidant activity (%AA), reflecting the inhibition of β‐carotene bleaching, was calculated using Equation (3):

(3) $$\%AA=\left[\frac{\left(A_{control\left(t\right)}-A_{sample\left(t\right)}\right)}{A_{control\left(t\right)}}\right]\;\times 100\;$$

where$A_{control(t)}$ and $A_{sample(t)}$ are the absorbances of the control and sample at time *t*, respectively.

#### 2.4.5 Superoxide Anion Scavenging Assay

The ability to scavenge superoxide anion radicals (O_2_•^−^) was determined using the riboflavin–light–NBT system [41]. The reaction mixture, with a total volume of 2.15 mL, contained 1 mL of sample, 0.5 mL of phosphate buffer (50 mM, pH 7.6), 0.3 mL of riboflavin (50 µM), 0.25 mL of phenazine methosulfate (PMS, 20 µM), and 0.1 mL of nitro blue tetrazolium (NBT, 5 mM).

The reaction was initiated by illuminating the mixture with fluorescent light for 20 min to generate superoxide radicals via riboflavin photoreduction. The absorbance of the resulting solution was then measured at 560 nm. Ascorbic acid served as the standard antioxidant. The superoxide radical scavenging activity (%) was calculated using Equation (4):

(4) $$\%Scavenging=\left[\frac{\left(A_{control}-A_{sample}\right)}{A_{control}}\right]\;\times 100$$

where $A_{control}$ and $A_{sample}$ are the absorbances of the control (without sample) and the test sample, respectively.

#### 2.4.6 Hydrogen Peroxide Scavenging Assay

The hydrogen peroxide (H_2_O_2_) scavenging capacity was evaluated according to the method of Ruch et al. [42]. Briefly, 0.1 mL of the sample was mixed with 0.9 mL of phosphate buffer (50 mM, pH 7.4). Then, 0.6 mL of a freshly prepared H_2_O_2_ solution (2 mM in the same buffer) was added. The mixture was vortexed and incubated at room temperature for 10 min. The absorbance was measured at 230 nm against a blank containing buffer without H_2_O_2_. The scavenging percentage was calculated using Equation (5):

(5) $$\%Scavenging=\left[\frac{\left(A_{control}-A_{sample}\right)}{A_{control}}\right]\;\times 100\;\;$$

where $A_{control}$ is the absorbance of the H_2_O_2_ solution without sample.

#### 2.4.7 Hydroxyl Radical Scavenging Assay

Hydroxyl radical (•OH) scavenging activity was determined using the deoxyribose degradation assay [43]. The reaction mixture (1.16 mL) contained 500 µL of 2‐deoxyribose (2.8 mM), 200 µL of phosphate buffer (50 mM, pH 7.4), 260 µL of an FeCl_3_‐EDTA solution (100 mM each, 1:1 v/v), 100 µL of H_2_O_2_ (200 mM), and 100 µL of sample.

After incubation at 37°C for 1 h, 1 mL each of trichloroacetic acid (2.8% w/v) and thio‐barbituric acid (1% w/v) were added. The mixture was heated in a boiling water bath for 15 min to develop the chromogen, and its absorbance was measured at 532 nm. The scavenging percentage was calculated using Equation (6):

(6) $$\%Inhibition=\left[\frac{\left(A_{control}-A_{sample}\right)}{A_{control}}\right]\;\times 100\;\;$$

where $A_{control}$ is the absorbance of the reaction mixture without the sample.

#### 2.4.8 Hemolysis Examination

The anti‐hemolytic activity of the ZnO/CuO@amoxicillin nanocomposite was evaluated using an oxidative stress‐induced model [44]. Briefly, washed human erythrocytes (O+ type) were suspended in PBS. For the assay, 40 µL of packed erythrocytes were incubated with 2 mL of nanocomposite suspension at various concentrations for 5 min at 37°C. Oxidative hemolysis was then induced by adding 40 µL of a 1:1 (v/v) mixture of FeCl_3_ (80 mM) and H_2_O_2_ (30 mM), followed by further incubation at 37°C for 1 h.

The samples were centrifuged at 700 × *g* for 10 min. The absorbance of the supernatant, indicative of released hemoglobin, was measured at 540 nm. Ascorbic acid was used as a positive control. The percentage of hemolysis was calculated using Equation (7):

(7) $$\%Hemolysis=\left(A_{sample}/A_{total}\right)\;\times 100\;$$

where $A_{sample}$ is the absorbance of the test supernatant and $A_{total}$ is the absorbance of the supernatant from erythrocytes lysed completely with distilled hemolysis control) water (100%

### 2.5 Anti‐Inflammatory Activity

#### 2.5.1 Egg Albumin Denaturation Assay

Anti‐inflammatory activity was evaluated using the egg albumin denaturation inhibition assay [45]. Briefly, a reaction mixture (5 mL total volume) was prepared with 0.2 mL of fresh hen egg albumin, 2.8 mL of phosphate‐buffered saline (PBS, pH 7.4), and 2 mL of the nanocomposite sample at various concentrations. The mixture was incubated at 37°C for 15 min and then heated at 70°C for 5 min to induce denaturation. After cooling to room temperature, the absorbance was measured at 660 nm. Diclofenac sodium served as the standard drug. The percentage inhibition of protein denaturation was calculated using Equation (8):

(8) $$\%Inhibition=\left[1-\left(\frac{\left(A_{sample}-A_{sample\;blank}\right)}{\left(A_{control}-A_{controlblank}\right)}\right)\right]\;\times 100\;$$

where $A_{sample}$ and $A_{control}$ are the absorbances of the test sample and the control (PBS instead of sample) after heating, respectively. $A_{sampleblank}$ and $A_{controlblank}$ are the corresponding absorbances of unheated mixtures.

#### 2.5.2 Human Serum Albumin (HSA) Denaturation Assay

The ability to inhibit the denaturation of human serum albumin (HSA) was assessed as described [46, 47]. A 1 mL aliquot of the sample was mixed with 1 mL of HSA solution (5% in PBS, pH 7.4). The mixture was incubated at 27°C for 15 min and then heated at 70°C for 10 min. After cooling, the absorbance was measured at 660 nm. Diclofenac sodium was used as the standard, and the inhibitory activity is expressed in mg diclofenac sodium equivalents per mg of sample.

### 2.6 Antidiabetic Activity

#### 2.6.1 α‐Glucosidase Inhibitory Activity

The α‐glucosidase inhibitory activity was evaluated using a microplate‐based method adapted from the literature [48]. Briefly, 50 µL of phosphate buffer (pH 6.8) was mixed with 10 µL of α‐glucosidase solution (1 U/mL) and 20 µL of the nanocomposite suspension at various concentrations in a 96‐well plate. The mixture was pre‐incubated at 37°C for 15 min. Then, 20 µL of the substrate p‐nitrophenyl‐α‐d‐glucopyranoside (p‐NPG, 5 mM) was added, and the reaction was allowed to proceed for 20 min at 37°C. The reaction was stopped by adding 50 µL of 0.1 M Na_2_CO_3_. The absorbance of the released p‐nitrophenol was measured at 405 nm. Acarbose was used as a positive control. The inhibition percentage was calculated, and the IC_50_ value was determined from a dose‐response curve.

#### 2.6.2 α‐Amylase Inhibitory Activity

The α‐amylase inhibitory activity was assessed according to a reported method [49]. In a 96‐well plate, 50 µL of phosphate buffer (100 mM, pH 6.8), 10 µL of α‐amylase solution (2 U/mL), and 20 µL of nanocomposite suspension were mixed and incubated at 37°C for 20 min. Then, 50 µL of soluble potato starch (1% w/v in buffer) was added as the substrate, and incubation continued for 30 min at 37°C. The reaction was terminated by adding 100 µL of dinitrosalicylic acid (DNS) reagent, followed by heating in a boiling water bath for 10 min. Aftercooling, the absorbance was measured at 540 nm. Acarbose served as the standard inhibitor. The inhibition percentage was calculated, and the IC_50_ value was derived.

### 2.7 Acetylcholinesterase (AChE) Inhibition Assay

AChE inhibitory activity was determined using a modified Ellman's method. Briefly, 200 µL of the sample was mixed with 100 µL of AChE enzyme solution and 200 µL of 0.1 M phosphate buffer (pH 8) containing 300 µL of 5,5’‐dithiobis (2‐nitrobenzoic acid) (DTNB). The mixture was incubated for 15 min at room temperature [49]. The enzymatic reaction was then initiated by adding 100 µL of acetylthiocholine iodide (ACTI) substrate.

The hydrolysis of ACTI by AChE produces thiocholine, which reacts with DTNB to form the yellow‐colored 5‐thio‐2‐nitrobenzoate anion (TNB^2^^−^). The increase in absorbance at 412 nm, monitored for 5–10 min, is proportional to the enzyme activity. A control (buffer in place of the sample) and appropriate blanks (enzyme or substrate omitted) were included in parallel [50].

The percentage inhibition of AChE was calculated using Equation (9):

(9) $$\%Inhibition=\left[1-\left(\frac{\left(A_{sample}-A_{sample\;blank}\right)}{\left(A_{control}-A_{controlblank}\right)}\right)\right]\;\times 100\;$$

where$A_{sample}$ and $A_{control}$ are the reaction absorbances with and without the inhibitor, respectively. $A_{sampleblank}$ and $A_{controlblank}$ are the corresponding absorbances of the nonenzymatic blanks.

### 2.8 Statistical Analysis

All biological experiments were performed in triplicate and repeated independently three times (*n* = 3). Results are expressed as mean ± standard deviation (SD). IC_50_ values were calculated from the dose‐response data using nonlinear regression analysis. Statistical comparisons between the ZnO/CuO@amoxicillin nanocomposite and the respective reference standards (acarbose, diclofenac sodium, ascorbic acid, and donepezil) were carried out using one‐way ANOVA followed by Tukey's post‐hoc multiple comparison test. For pairwise comparisons, an unpaired Student's *t*‐test was applied when appropriate. A *p*‐value of less than 0.05 was considered statistically significant.

## 3 Results and Discussion

### 3.1 Characterization

#### 3.1.1 UV–vis Analysis

**FIGURE 1:**
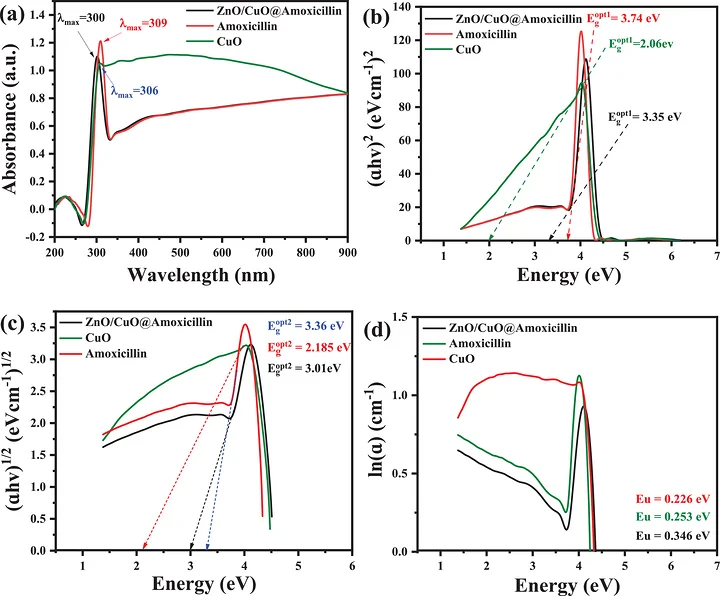
UV–vis absorption spectra and optical band‐structure parameters of ZnO/CuO@amoxicillin compared with CuO NPs and amoxicillin: (a) Absorption spectra, (b–d) Tauc plots for band‐gap estimation, and (d) Urbach analysis for tail‐state/disorder evaluation.

Figure 1a shows that ZnO/CuO@amoxicillin exhibits a broader and more intense absorption band than CuO or amoxicillin, with a stronger extension into the visible region. This broadening indicates interfacial electronic coupling in the hybrid, arising from the ZnO/CuO heterojunction and charge‐transfer interactions with Amoxicillin, as commonly observed in oxide–drug systems where interfacial bonding modifies the electronic structure [51].

Tauc plots (Figure 1b–d) yield an optical band gap of *E*_g_≈3.30–3.36 eV for ZnO/CuO@amoxicillin, intermediate between ZnO (≈3.7–3.8 eV) and CuO (≈2.0–2.2 eV). This shift supports band‐structure tuning driven by formation of the p–n ZnO/CuO junction and interfacial (surface/defect) states introduced upon hybridization, consistent with prior ZnO/CuO reports [52].

Urbach analysis (Figure 1d) gives Eu = 0.346 eV for ZnO/CuO@amoxicillin, compared with 0.253 eV for Amoxicillin and 0.226 eV for CuO NPs. The higher Eu indicates increased electronic disorder and a greater density of tail states, likely associated with defects/oxygen vacancies and heterointerface effects, enabling lower‐energy transitions in the 1.5–3 eV range. These tail states can facilitate charge separation and suppress electron–hole recombination, strengthening surface redox activity, which aligns with the experimentally observed improvements in radical scavenging (DPPH, ABTS, FRAP, H_2_O_2_, and superoxide), reduced IC_50_, and stronger α‐amylase/α‐glucosidase inhibition versus CuO NPs or amoxicillin alone. The same electronic/defect‐rich surface environment is consistent with improved protection against protein denaturation and oxidative hemolysis through more effective ROS interception at reactive surface sites [51].

Compared with ZnO/CuO nanocomposites reported for enhanced functional performance, integrating amoxicillin provides added bioactivity while reinforcing interfacial electronic modulation, yielding a synergistic multifunctional response. Overall, the UV–vis, band‐gap, and Urbach outcomes coherently explain the enhanced functional performance of ZnO/CuO@amoxicillin and support its potential as a multifunctional therapeutic platform relevant to multidrug‐resistant pathogens.

#### 3.1.2 Fourier‐Transform Infrared Spectroscopy (FTIR)

**FIGURE 2:**
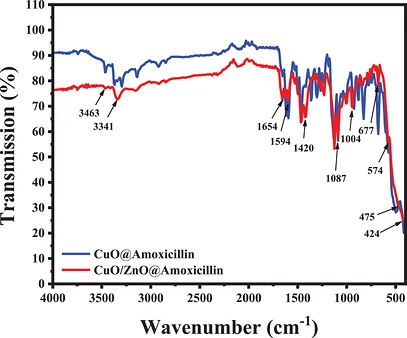
FTIR spectral signatures of CuO@Amoxicillin and CuO/ZnO@Amoxicillin showing Amoxicillin‐related functional groups and the metal–oxygen fingerprint region (Cu─O and Zn─O bands).

FTIR spectroscopy was used to identify functional groups and probe interfacial interactions in CuO@amoxicillin and ZnO‐doped CuO/ZnO@amoxicillin. As shown in Figure 2, the CuO/ZnO@amoxicillin spectrum exhibits additional bands and noticeable wavenumber shifts relative to the parent material, indicating chemical interactions associated with hybrid formation and drug immobilization [8].

In the high‐wavenumber region, bands at 3463 and 3341 cm−^1^ are assigned to O─H stretching of adsorbed surface water/hydroxyls and N─H stretching of the amine group in amoxicillin, suggesting an increased hydroxylated surface after ZnO incorporation. Characteristic Amoxicillin‐related vibrations appear at 1654 cm^−^^1^ (C═O, amide I) and 1594 cm^−^^1^ (aromatic C═C and/or amide II N─H bending), while the band at 1420 cm−^1^ corresponds to asymmetric stretching of COO^−^, supporting drug stabilization/anchoring on the nanoparticle surface. Additional bands at 1087 and 1004 cm^−^^1^ are attributed to C─O and C─N stretching vibrations, consistent with the molecular framework of amoxicillin [8, 53].

In the metal–oxygen fingerprint region, Cu─O lattice vibrations are evidenced by bands at 677 and 574 cm−^1^. The emergence of a distinct band at 475 cm^−^^1^ confirms Zn─O stretching, providing direct evidence for the presence of the ZnO phase within the hybrid material. Moreover, the band at 424 cm^−^^1^, together with the feature near 942 cm^−^^1^ (possibly related to deformation modes), supports oxide–oxide interaction and the formation of a CuO/ZnO hybrid nanostructure upon doping [54, 55, 56].Overall, the combined appearance of new bands and systematic shifts substantiates successful ZnO incorporation and interfacial bonding (e.g., hydrogen bonding and coordination), which are expected to enhance the surface chemistry and functional performance of CuO/ZnO@Amoxicillin for downstream applications [57, 58].

#### 3.1.3 XRD Pattern Analysis

**FIGURE 3:**
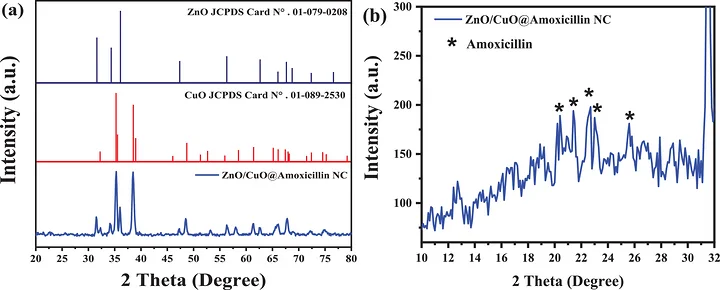
XRD pattern of the ZnO/CuO@Amoxicillin nanocomposite showing (a) the coexistence of wurtzite ZnO and monoclinic CuO phases and (b) Amoxicillin Phase.

The XRD pattern of ZnO/CuO@amoxicillin (Figure 3) confirms the coexistence of wurtzite ZnO and monoclinic CuO, together with a weak diffuse background attributable to the amorphous organic component. The ZnO reflections can be indexed to the characteristic wurtzite planes (e.g., (100), (002), (102), (110), (200), (004), and (202)), indicating that the hexagonal lattice is preserved within the hybrid. This phase assignment is consistent with standard ZnO reference data widely reported using PDF/JCPDS 01‐079‐0208 [59, 60].​

CuO is identified by its characteristic monoclinic reflections in the mid‐angle region (notably near 2*θ* ≈ 35°–39°, 48°–49°, and ∼58°–59°), which remain distinguishable despite partial overlap with ZnO peaks. Such peak overlap is expected in mixed‐oxide systems and can be resolved by careful indexing against the standard CuO reference card (PDF/JCPDS 01‐089‐2530) [61].​

Crystallite size was estimated from line broadening using the Scherrer approach across multiple reflections, yielding nanoscale domains on the order of ∼10–12 nm. Because peak broadening may include both finite‐size and microstrain contributions, Williamson–Hall analysis provides a standard route to separate these effects and strengthen the reliability of size–strain interpretation in nanocrystalline materials [62].​

Overall, the agreement of the experimental diffractogram with ZnO (PDF/JCPDS 01‐079‐0208) and CuO (PDF/JCPDS 01‐089‐2530) reference patterns supports robust phase identification and structural integrity of the hybrid inorganic framework. The confirmed biphasic oxide crystallinity (ZnO/CuO) provides a structural basis for enhanced surface‐mediated reactivity in multifunctional applications, while the presence of an amorphous contribution is consistent with drug incorporation in the composite [59, 61].

The figure 3 (b) restricted to the 2*θ* range of 10°–32° clearly reveals the characteristic peaks of amoxicillin, which were not visible in the full scan due to the dominant diffraction signals of ZnO and CuO. This observation confirms the successful incorporation of the drug into the nanocomposite. Similar findings have been reported in previous studies, where the crystalline signals of organic molecules were masked by strong inorganic phases but became evident under focused scans or magnified regions of the diffractogram [63, 64]. These results support the stability of amoxicillin within the ZnO/CuO matrix and validate its presence as indicated by the marked peaks.

The high crystallinity and nanoscale grain size are directly linked to enhanced surface reactivity, which explains the strong biological activities observed in this study [65]. The composite exhibited significant AChE inhibition, anti‑inflammatory activity (egg albumin denaturation), hemolysis protection, β‑carotene bleaching, and antidiabetic effects (α‑amylase and α‑glucosidase inhibition). Furthermore, antioxidant assays (DPPH, ABTS, hydroxyl radical, superoxide, hydrogen peroxide scavenging, FRAP, and TAC) confirmed the role of crystalline ZnO/CuO domains in promoting electron transfer and radical neutralization. These findings demonstrate that the structural integrity revealed by XRD is consistent with the biological performance of the composite, supporting its potential in biomedical and environmental applications.

#### 3.1.4 SEM analysis

**FIGURE 4:**
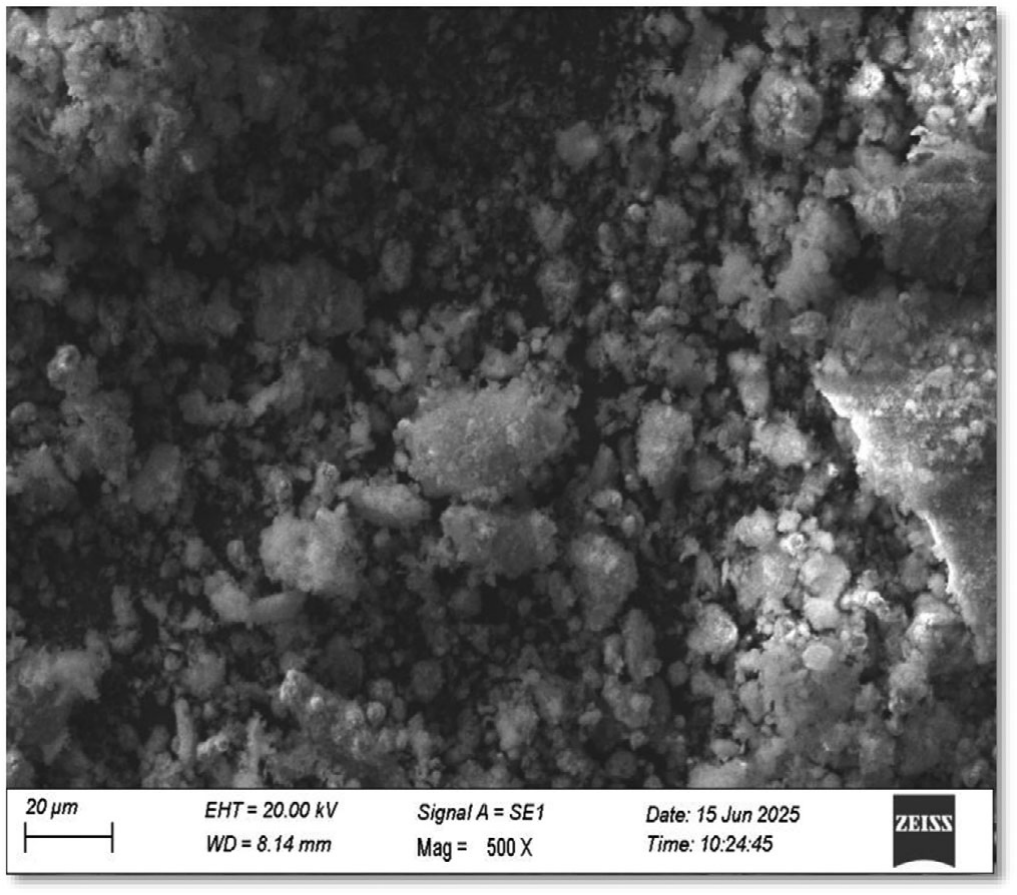
SEM micrograph of the ZnO/CuO@Amoxicillin nanocomposite revealing surface morphology and particle aggregation.

The SEM micrograph of ZnO/CuO@amoxicillin (Figure 4) displays a heterogeneous surface morphology comprising irregularly shaped particles with varied sizes and agglomeration states. High‐magnification imaging (500×) reveals densely packed grains with rough textures and observable porosity, indicating a high specific surface area. The particle size distribution spans from fine nanometric domains to larger secondary clusters, a hierarchical arrangement consistent with the crystallite size trends (∼11 nm) determined from XRD, where primary nanocrystals tend to aggregate into microscale assemblies [66, 67].​

The SEM images suggest surface roughness and possible porous‐like morphology, which may be advantageous for biological applications by increasing the contact area available for molecular adsorption and interaction. Such morphological features—particularly the irregularity and intergranular voids—may contribute to the observed enzyme inhibition and radical‐scavenging activity, although quantitative confirmation of porosity and surface area requires BET analysis (AChE, α‐amylase, and α‐glucosidase) and radical‐scavenging activity (DPPH, ABTS, FRAP, and superoxide) by facilitating substrate access and active site exposure. Furthermore, the absence of extensive sintering or smooth fused surfaces suggests that the organic phase (Amoxicillin) is reasonably distributed within or on the inorganic matrix, without compromising the particulate nanostructure [68, 69].​

Imaging conditions (WD = 8.14 mm, EHT = 20.00 kV, SE1 mode) provided sufficient depth of field and edge contrast to resolve these topographical details clearly. Overall, SEM analysis corroborates the XRD‐derived structural model, confirming the formation of a nanostructured, porous, and morphologically integral hybrid system well‐suited for biomedical and environmental deployment [25, 70].​

### 3.2 In Vitro Biological Activities of ZnO/CuO@Amoxicilline Nanocomposite

#### 3.2.1 Antidiabetic Activities

##### 3.2.1.1 α‐Amylase Inhibition

**FIGURE 5:**
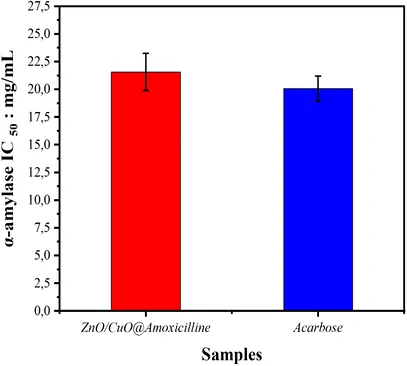
α‐Amylase inhibitory activity (IC_50_) of ZnO/CuO@Amoxicillin nanoparticles compared with acarbose.

The ZnO/CuO@amoxicillin nanocomposite exhibited α‐amylase inhibitory activity with an IC_50_ value of 21.57 ± 1.68 mg/mL. This was comparable to that of the reference inhibitor acarbose (20.07 ± 1.12 mg/mL), with no statistically significant difference observed (*p* > 0.05). These results suggest that the nanocomposite effectively retards starch hydrolysis, supporting its potential role in managing postprandial hyperglycemia (Figure 5). This inhibition underlines the potential of nanoparticles to manage postprandial hyperglycemia by retarding enzymatic conversion of starch into maltose and glucose. Mechanistically, the high surface reactivity of CuO and ZnO produces electrostatic and hydrogen bond interactions with the active site residues of α‐amylase, probably inducing conformational distortions that reduce catalytic efficiency [71]. The decoration with amoxicillin adds further stabilization via hydrophobic and polar interactions, leading to increased inhibitory effect [72]. In addition, ROS generated by the nanoparticles can induce subtle oxidative modifications in thiol groups or metal‐binding residues of the enzyme, which further reduces its activity [73]. As compared to previous studies, including trimetallic Se/ZnO/CuO nanoalloys of Amin et al. [71] having α amylase IC_50_ of 69.3 µg/mL, our system operates at higher concentrations but has the advantage of multifunctionality by simultaneously providing antimicrobial activity [74]. Moreover, plant‐mediated ZnO or CuO NPs typically exhibited IC_50_ values in the range of 25–35 mg/mL for inhibition of α‐amylase, suggesting that bimetallic CuO/ZnO hybrid combined with amoxicillin conferred comparable or better efficacy, emphasizing the synergy of dual metal oxides and surface functionalization [75].

The inhibitory effect is likely attributed to adsorption of the ZnO/CuO@Amoxicillin nanocomposite onto the enzyme surface, where Cu^2^^+^ and Zn^2^^+^ ions interact with key catalytic residues (e.g., Asp and Glu) via electrostatic and coordination interactions, leading to localized conformational alterations that reduce catalytic efficiency [76].

Furthermore, amoxicillin functionalization introduces additional hydrogen bonding and hydrophobic interaction sites, enhancing binding affinity and stabilizing the enzyme–inhibitor complex compared to bare metal oxides [77].

Overall, this is a multitasking inhibitory profile and positions nanoparticles as promising candidates toward integrated therapeutic strategies targeting hyperglycemia and microbial infections simultaneously.

##### 3.2.1.2 α‐Glucosidase Inhibition

**FIGURE 6:**
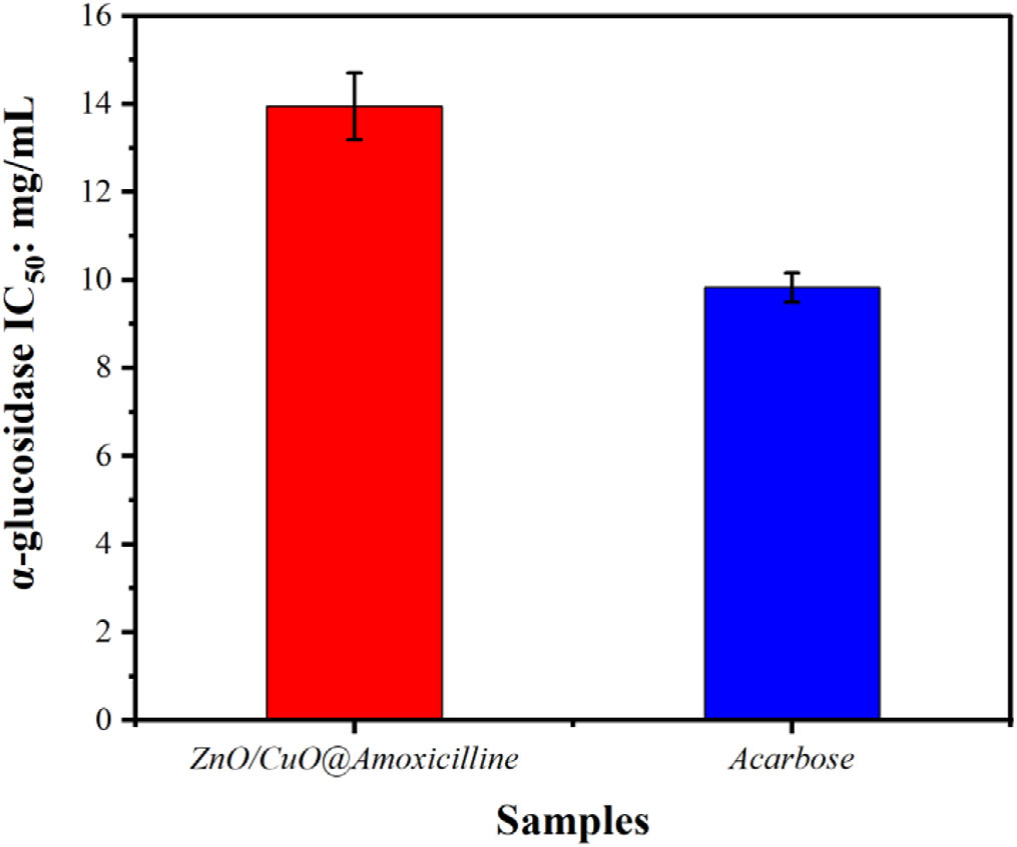
α‐Glucosidase inhibitory activity (IC_50_) of ZnO/CuO@Amoxicillin nanoparticles compared with acarbose.

The nanocomposite showed α‐glucosidase inhibitory activity with an IC_50_ of 13.95 ± 0.76 mg/mL, which was slightly higher than that of acarbose (9.83 ± 0.33 mg/mL; *p* < 0.05). Despite this difference, the activity remains in a promising range for a multifunctional nanomaterial (Figure 6). This dual inhibition is critical in mitigating postprandial glucose spikes [75]. The nanoparticles’ surface chemistry allows them to occupy or interact closely with the enzyme's active site, hindering substrate access, while the amoxicillin coating enhances the stability and affinity of this interaction through polar contacts with key residues [78]. The synergy between CuO and ZnO further strengthens enzyme modulation compared to single‐metal nanoparticles, consistent with literature showing that mixed‐metal oxide systems outperform their individual components [79]. Relative to Amin et al., which reported αglucosidase IC50 of 40.8 µg/mL for Se/ZnO/CuO nanoalloys, our system requires higher concentrations but simultaneously integrates antibacterial function, which is not the focus of the trimetallic system [80].

The inhibitory mechanism is likely driven by the adsorption of ZnO/CuO@Amoxicillin nanoparticles onto the enzyme surface, where Zn^2^^+^ and Cu^2^^+^ ions can interact with catalytic residues through coordination and electrostatic interactions, thereby blocking access of oligosaccharide substrates to the active site. Additionally, the Amoxicillin coating enhances binding specificity via polar and hydrogen‐bonding interactions with amino acid residues surrounding the active pocket, leading to stabilization of an inactive enzyme conformation [81]. The bimetallic CuO/ZnO structure further promotes synergistic effects by increasing surface reactivity and facilitating multivalent interactions, which are more effective than monometallic systems in perturbing enzyme function [82]. Comparatively, plant‐derived ZnO or CuO nanoparticles often achieved IC_50_ values of 15–22 mg/mL, placing our bimetallic antibiotic‐functionalized nanoparticles in a similar or slightly superior activity range [83]. The concurrent targeting of αglucosidase, along with α amylase, underlines the therapeutic potency of ZnO/CuO@Amoxicillin NPs as a comprehensive, multifunctional agent capable of managing postprandial hyperglycemia while providing antimicrobial protection, therefore offering an advantage over conventional monotherapy or single‐metal nanoparticles [77].

#### 3.2.2 Anti‐Inflammatory Activity

##### 3.2.2.1 Inhibition of Egg Albumin Denaturation Assay

**FIGURE 7:**
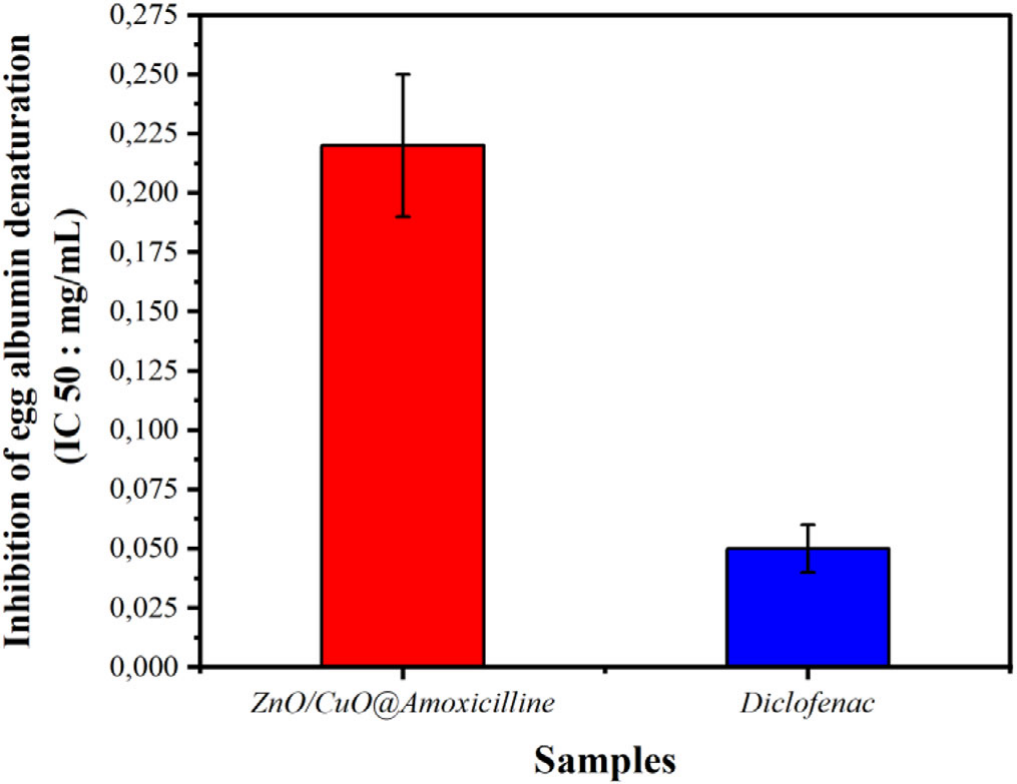
Inhibition of egg albumin denaturation by ZnO/CuO@Amoxicillin nanoparticles compared with diclofenac (IC_50_ values).

In the egg albumin denaturation assay, the ZnO/CuO@Amoxicillin nanocomposite demonstrated an IC_50_ of 0.22 ± 0.03 mg/mL. This value was statistically comparable to diclofenac sodium (*p* ≤ 0.05), indicating notable protein stabilization capacity under heat‐induced stress (Figure 7). In a mechanistic perspective, highly reactive surfaces of both CuO and ZnO nanoparticles interact by means of hydrogen bonding and electrostatic contacts with polar and charged amino acid residues of egg albumin, thereby preserving its tertiary structure [84]. An amoxicillin coating further enhances these effects by adding hydrophobic interactions‐effectively wrapping a protective sheath around the protein [85]. Unlike enzyme inhibition mechanisms, this effect is primarily governed by structural preservation rather than active‐site interaction, highlighting a distinct mode of bioactivity [86]. Additionally, controlled nano–bio interfacial effects may induce subtle structural rearrangements that favor a more stable protein configuration under stress conditions, without causing irreversible oxidative damage [87]. Moreover, mild ROS generated at the nanoparticle interface may cause a subtle oxidation of sensitive amino acids, thereby forcing structural rearrangements that paradoxically result in protein stabilization rather than its degradation [88]. Indeed, compared to their single‐metal oxide analogs or free amoxicillin, this nanostructure leads to a synergistic effect toward better protection of proteins [89]. The multifunctional design allows the simultaneous exertion of antimicrobial and antidiabetic effects, thus placing these nanoparticles as promising versatile therapeutic candidates in inflammation‐prone environments, while concurrently mitigating potential oxidative protein damage [90].

##### 3.2.2.2 Human Serum Albumin (HSA) Denaturation

**FIGURE 8:**
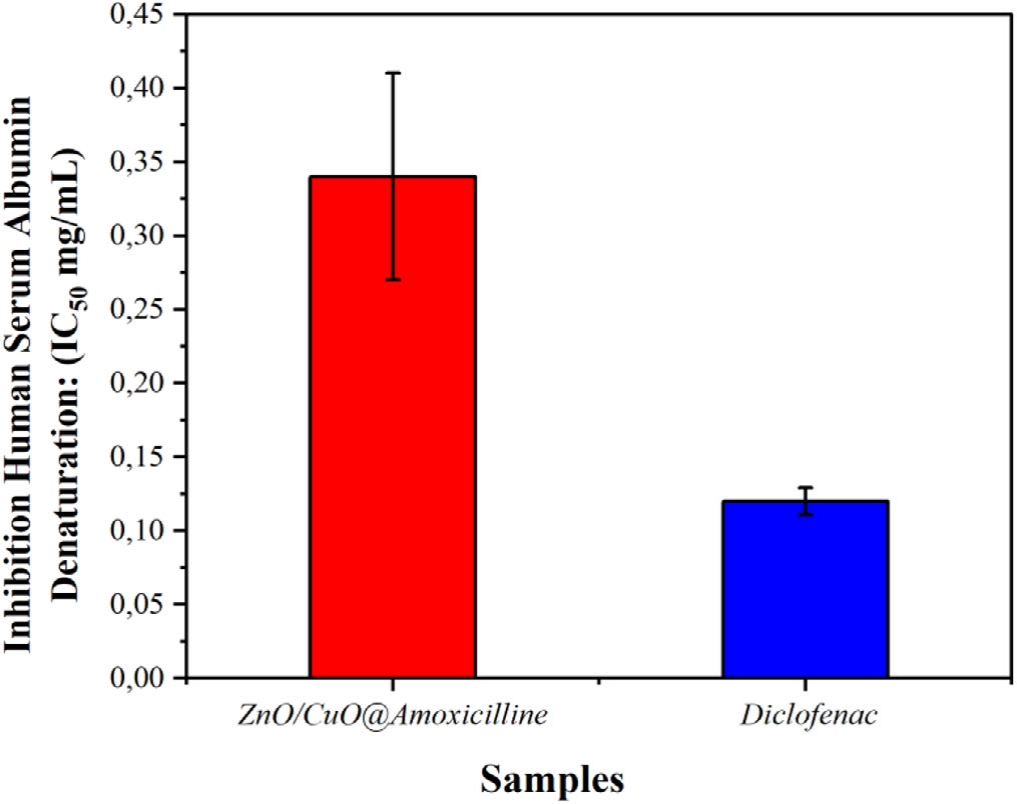
Inhibition of human serum albumin (HSA) denaturation by ZnO/CuO@Amoxicillin nanoparticles compared with diclofenac (IC_50_ values).

The ZnO/CuO@amoxicillin nanocomposite inhibited heat‐induced denaturation of human serum albumin with an IC_50_ value of 0.34 ± 0.07 mg/mL. This value was slightly higher than that of the reference drug diclofenac sodium (IC_50_ = 0.12 ± 0.009 mg/mL), with a statistically significant difference (*p* < 0.05). Nevertheless, the observed activity indicates a notable capacity to stabilize a physiologically relevant human protein (Figure 8). The anti‐inflammatory mechanism is via both structural and redox‐mediated pathways: the surfaces of the nanoparticles present electrostatic, coordination, and hydrogen‐bond interactions toward HSA, anchoring the latter and reducing its conformational flexibility under heat stress [15]. The anti‐denaturation effect of ZnO/CuO@amoxicillin is primarily attributed to the formation of a stable nano–protein corona, in which the nanoparticle surface interacts dynamically with HSA, limiting conformational mobility under thermal stress [8]. Specifically, Cu^2^^+^ and Zn^2^^+^ ions can establish coordination interactions with electron‐donating groups (e.g., imidazole, carboxylate, and thiol residues), while electrostatic and hydrogen bonding interactions further anchor the protein structure, reducing unfolding propensity. The amoxicillin shell enhances this stabilization by reinforcing the nano–bio interface through combined hydrophobic and polar interactions, thereby acting as a protective molecular layer that preserves the native conformation of HAS [8].Additionally, the outer shell made up of amoxicillin acts in a synergistic way to strengthen the nanoparticle‐protein interface through hydrophobic and polar contacts, therefore effectively preventing unfolding [25]. In addition, controlled ROS generation at the interface may transiently oxidize critical thiol or methionine residues, which would eventually favor intramolecular crosslinks that enhance stability without causing irreversible damage. Unlike some literature reports on ciprofloxacin‐loaded CuO or Ag nanoparticles, which presented protein stabilization and redox modulation properties, the CuO/ZnO@Amoxicillin system adds to these an antimicrobial role and a metabolic regulatory function [91]. Thus, such a dual action‐direct protein protection and redox environmental modulation‐supports a multifunctional therapeutic potential against inflammatory conditions linked to infection and oxidative stress [92].

#### 3.2.3 Inhibition of Acetylcholinesterase (AChE) Activity

**FIGURE 9:**
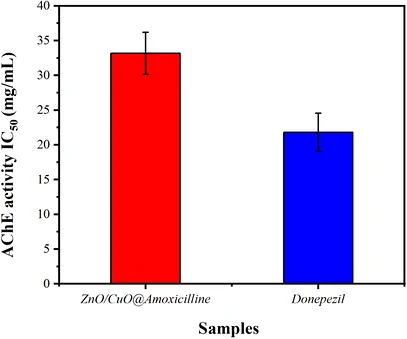
Acetylcholinesterase (AChE) inhibitory activity of ZnO/CuO@Amoxicillin nanoparticles compared with Donepezil (IC_50_ values).

The ZnO/CuO@amoxicillin nanocomposite inhibited acetylcholinesterase activity with an IC_50_ value of 33.16 ± 3.02 mg/mL. This value was significantly higher than that of the reference drug donepezil (21.80 ± 2.74 mg/mL, *p* < 0.05), indicating moderate inhibitory potency (Figure 9). These values may reflect a moderate yet statistically significant potential to modulate cholinergic signaling, which is particularly important in neurodegenerative diseases such as Alzheimer's disease [93]. Mechanistically, this inhibition likely arises due to multiple complementary interactions: the metal oxide core (CuO/ZnO) could, via electrostatic and coordination interactions, bind to the active gorge of AChE and alter the conformation of catalytic residues [94]. Simultaneously, the amoxicillin moieties on the nanoparticle surface might create additional hydrogen bonding and hydrophobic interactions, stabilizing the nanoparticle‐enzyme complex and blocking access by substrates [95].

Additionally, the nanoparticle surface can adsorb onto the enzyme through electrostatic interactions, given the positively charged nature of many metal oxide surfaces at physiological pH and the presence of negatively charged residues around the active gorge. The amoxicillin molecules functionalized on the surface may further enhance inhibition by forming hydrogen bonds with polar residues (such as tyrosine or tryptophan) and hydrophobic interactions near the peripheral anionic site, thereby stabilizing the nanoparticle–enzyme complex and restricting substrate (acetylcholine) access to the catalytic gorge [96]. Similar surface adsorption and metal ion‐mediated mechanisms have been reported for ZnO and CuO nanoparticles in previous studies, where Zn^2^^+^ release or direct binding altered AChE secondary structure and inhibited enzymatic function. The hybrid ZnO/CuO structure combined with amoxicillin functionalization appears to provide a synergistic contribution through improved surface reactivity and additional binding sites [97]. Indeed, comparable studies on different metal oxide nanoparticles like CuO, ZnO, and Ag NPs have shown similar surface‐mediated binding as a mechanism for AChE inhibition, reinforcing the importance of the nanoparticle's surface chemistry in modulating enzyme activities [98]. Less potent than donepezil, the multi‐functional CuO/ZnO@Amoxicillin system brings into play enzyme inhibition together with its anti‐microbial, antioxidant, and anti‐inflammatory actions, therefore offering an integrated nano‐therapeutic platform for neuroinflammation and oxidative‐stress‐related disorders [99].

#### 3.2.4 Antioxidant Capacity

##### 3.2.4.1 Acid/β‐Carotene Bleaching Assay

**FIGURE 10:**
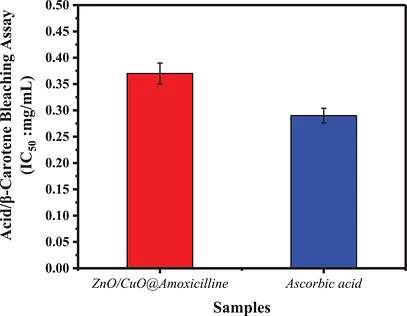
Acid/β‐carotene bleaching assay of ZnO/CuO@Amoxicillin nanoparticles compared with ascorbic acid (IC_50_ values).

The ZnO/CuO@amoxicillin nanocomposite showed β‐carotene bleaching inhibitory activity with an IC_50_ value of 0.37 ± 0.02 mg/mL. This value was slightly higher than that of ascorbic acid (0.29 ± 0.14 mg/mL), with a statistically significant difference (p < 0.05) (Figure 10). It appears that this effectively inhibits lipid peroxidation and protects β‐carotene from oxidative degradation under conditions of stress, therefore reflecting the capacity of the nanoparticles to scavenge active radicals in a lipid‐rich environment [8]. Mechanistically, the metal oxide core of CuO/ZnO acts by electron transfer and radical quenching to generate stable surface‐bound radicals that prevent further oxidative chain reactions [100]. Additionally, the high surface area of the nanocomposite enhances adsorption of reactive species, increasing the probability of radical neutralization at the nano–lipid interface. The amoxicillin coating enhances the structural stability of the nanoparticles by offering additional hydrogen bonding and electron‐donating sites that amplify radical‐scavenging efficiency [101]. Furthermore, the organic shell may improve dispersion of the nanocomposite within the emulsion system, promoting more effective interaction with lipid radicals [102]. The amoxicillin coating enhances the structural stability of the nanoparticles by offering additional hydrogen bonding/electron donation sites that amplify radical‐scavenging efficiency. Similar trends have been recorded in hybrid metal oxide nanoparticles, including ciprofloxacin‐loaded CuO or Ag NPs displaying enhanced lipid peroxidation inhibition via synergistic surface and drug‐mediated mechanisms [16]. Two functions are combined in one: the direct neutralization of radicals along with antimicrobial and anti‐inflammatory action positions CuO/ZnO@Amoxicillin nanoparticles as plausible candidates for mitigating oxidative stress in complex biological systems [103].

##### 3.2.4.2 Hemolysis Examination

**FIGURE 11:**
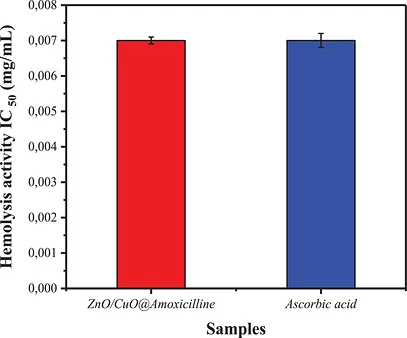
Hemolysis activity of ZnO/CuO@Amoxicillin nanoparticles compared with ascorbic acid (IC_50_ values). Data are presented as mean ± SD (*n* = 3). Statistical analysis was performed using one‐way ANOVA followed by Tukey's multiple comparison test. Different symbols/letters indicate statistically significant differences (*p* < 0.05).

The ZnO/CuO@Amoxicillin nanocomposite exhibited a protective effect against H_2_O_2_/FeCl_3_‐induced oxidative hemolysis in human erythrocytes, with an IC_50_ value of 0.007 ± 0.00 mg/mL. The percentage of hemolysis decreased progressively with increasing nanocomposite concentration, from 71.44% at 0.004 mg/mL to 29.00% at 0.010 mg/mL, indicating a concentration‐dependent protective effect (Table 1). This assay evaluates the protective effect of the nanocomposite against oxidative stress‐induced hemolysis rather than its intrinsic hemolytic potential. The IC_50_ value was comparable to that of the reference antioxidant ascorbic acid (0.007 ± 0.0002 mg/mL, *p* > 0.05) (Figure 11).

**TABLE 1:** Percentage of H_2_O_2_/FeCl_3_‐induced hemolysis in human erythrocytes treated with ZnO/CuO@Amoxicillin nanocomposite and ascorbic acid at different concentrations.

Concentration (mg/mL)	ZnO/CuO@Amoxicillin (% Hemolysis)	Ascorbic Acid (% Hemolysis)
Control	100.00	100.00
0.004	71.44	71.00
0.006	58.00	57.00
0.008	42.00	43.00
0.010	29.00	31.00

In this model, erythrocytes are exposed to a pro‐oxidant environment that generates ROS, particularly hydroxyl radicals through Fenton chemistry, which can induce lipid peroxidation of the erythrocyte membrane and ultimately lead to hemolysis [104]. The marked reduction in hemolysis observed with increasing nanocomposite concentration suggests effective protection against oxidative membrane damage. This activity may be associated with the combined physicochemical properties of the ZnO/CuO@amoxicillin nanocomposite. The CuO/ZnO core may contribute to ROS modulation by interacting with reactive oxygen species and reducing oxidative damage to membrane components. However, since the unmodified ZnO/CuO nanocomposite was not included as a control, the specific contribution of amoxicillin functionalization to the observed anti‐hemolytic activity cannot be determined [105, 106].

Unlike chemical antioxidant assays, this cell‐based model provides a physiologically relevant assessment of protection against oxidative membrane damage, where the preservation of erythrocyte membrane integrity is a key endpoint [107]. The protective effect of the ZnO/CuO@amoxicillin nanocomposite may involve stabilization of the erythrocyte membrane and attenuation of oxidative damage. In addition, interactions with the Fe^3^^+^/Fe^2^^+^ redox system may contribute to limiting the formation of highly reactive hydroxyl radicals (•OH) under the experimental conditions [107]. Compared with previously reported metal oxide nanoparticles, the ZnO/CuO@amoxicillin nanocomposite demonstrated promising protective activity against oxidative stress‐induced hemolysis, in addition to its antioxidant, antimicrobial, and anti‐inflammatory properties [108]. Overall, these findings indicate that the nanocomposite can mitigate oxidative membrane damage in a physiologically relevant cellular model, supporting its potential as a multifunctional bioactive nanomaterial [109].

##### 3.2.4.3 Total Antioxydant Capacity (TAC)

**FIGURE 12:**
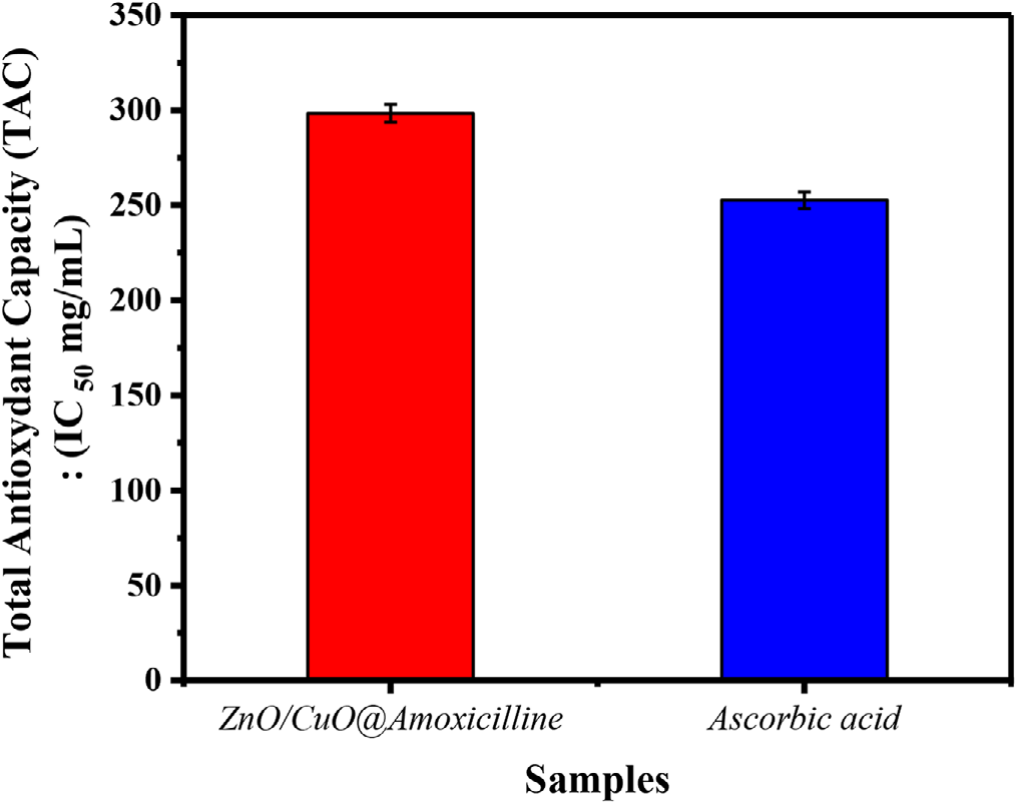
Total antioxydant capacity (TAC) of ZnO/CuO@Amoxicillin nanoparticles compared with ascorbic acid (IC_50_ values).

The total antioxidant capacity of the ZnO/CuO@amoxicillin nanocomposite was 298.27 ± 4.70 mg ascorbic acid equivalents per gram, which was significantly higher than that of the ascorbic acid standard (252.63 ± 4.37 mg/g, p < 0.01) (Figure 12). This indicates the strong capacity of the nanoparticles to scavenge a wide array of free radicals/reactive species, reflecting a wider redox modulation potential [16]. Mechanistically explained, the metal oxide core, CuO/ZnO, would enhance electron donation to reactive species, thus effectively terminating the radical chain reaction [110]. This is further assisted by an amoxicillin shell that increases surface reactivity by offering more electron‐rich sites and thereby stabilizing the generated radicals [111]. This dynamic redox cycling contributes to sustained antioxidant activity rather than a single‐step radical quenching process. The amoxicillin shell further enhances surface reactivity by providing additional electron‐rich and polar functional groups, stabilizing intermediate radical species [112].Such dual‐layer architecture has caused not only radical scavenging amplification but also the stabilization of the nanoparticle structure, avoiding its possible premature aggregation and thus assuring antioxidant performance [82, 113]. It appears that when these data are considered against previously published studies on ciprofloxacin‐ or plant‐mediated metal oxide nanoparticles, a similar synergistic TAC enhancement may be derived, thus showing the dominance of hybrid nanostructure over single‐component systems for broad‐spectrum protection against oxidative damage [114, 115]. This cooperative effect between the inorganic core and organic shell underlies the superior total antioxidant capacity compared to single‐component systems [112]. Overall, these results permit CuO/ZnO@Amoxicillin nanoparticles to be regarded as a class of multifunctional nanotherapeutics that could integrate antioxidant, antimicrobial, and anti‐inflammatory functions within one highly effective platform [16].

##### 3.2.4.4 Ferric Reducing Antioxidant Power (FRAP)

**FIGURE 13:**
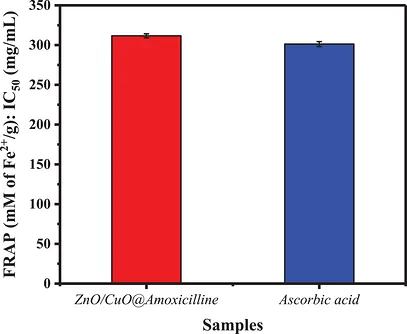
Ferric reducing antioxidant power capacity of ZnO/CuO@Amoxicillin nanoparticles compared with ascorbic acid (IC_50_ values).

The ferric reducing antioxidant power of the ZnO/CuO@Amoxicillin nanocomposite was 311.67 ± 2.53 mM Fe^2^^+^/g, which was slightly higher than that of the ascorbic acid standard (301.26 ± 3.08 mM Fe^2^^+^/g, *p* < 0.05) (Figure 13) [16]. This is mechanistically explained by the highly reactive surfaces of both CuO and ZnO, capable of transferring electrons directly to the oxidizing species, while the lattice defects and oxygen vacancies within the core of the metal oxides further allow the redox cycling and enhance the overall reducing power [114]. Interestingly, when compared with plant‐mediated or synthetic metal oxide nanoparticles from previous studies, the FRAP values were in a comparatively lower range; the hybrid nanoparticles have shown increased electron‐transfer efficiency in this study, most probably due to synergistic effects of the two metal oxides and optimized surface area [116]. Unlike lipid peroxidation or cellular antioxidant assays, the FRAP mechanism is dominated by direct reduction of oxidant species in a homogeneous system, highlighting a distinct pathway of antioxidant action. This strong reducing power complements other antioxidant mechanisms, reinforcing the nanocomposite's capacity to counteract oxidative stress through multiple redox pathways [117].This redox activity not only maintains the stability of free radicals but also complements other antioxidant activities, such as radical scavenging and lipid peroxidation inhibition, thus placing CuO/ZnO nanoparticles among highly versatile agents for mitigating oxidative stress in biological systems [118].

##### 3.2.4.5 Hydrogen Peroxide (H_2_O_2_) Scavenging Activity

**FIGURE 14:**
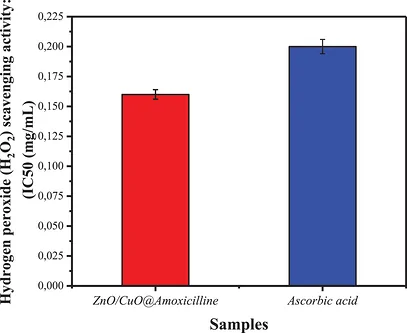
Hydrogen peroxide (H_2_O_2_) scavenging activity of ZnO/CuO@Amoxicillin nanoparticles compared with ascorbic acid (IC_50_ values). Data are presented as mean ± SD (*n* = 3). Statistical analysis was performed using one‐way ANOVA followed by Tukey's multiple comparison test. Different symbols/letters indicate statistically significant differences (*p* < 0.05).

The ZnO/CuO@Amoxicillin nanocomposite exhibited hydrogen peroxide scavenging activity with an IC_50_ value of 0.16 ± 0.04 mg/mL. This value was slightly lower than that of ascorbic acid (0.20 ± 0.00 mg/mL, *p* < 0.05), indicating higher scavenging efficiency (Figure 14). This assay reflects the capacity of the nanoparticles to prevent oxidative damage mediated by hydrogen peroxide, considered a key reactive oxygen species involved in lipid peroxidation and cellular injury [119]. From a mechanistic perspective, the redox‐active surfaces of CuO and ZnO will facilitate electron transfer to H_2_O_2_ and its further transformation into harmless water and oxygen. Similarly, structural stability due to surface coatings increases the persistence of this effect [120]. The presence of Cu^2^^+^/Cu^+^ redox couples may enable Fenton‐like or peroxidase‐mimetic activity, accelerating H_2_O_2_ decomposition while preventing accumulation of harmful intermediates. Additionally, surface defects and oxygen vacancies within the metal oxide lattice enhance adsorption of H_2_O_2_ molecules, increasing reaction efficiency at the nanoparticle interface. The amoxicillin coating further contributes by improving nanoparticle dispersion and stabilizing reactive surface sites, ensuring sustained scavenging activity [121].

Compared with previous studies on metal oxide nanoparticles (e.g., CuO, ZnO, and hybrid metal oxides), it is in agreement that nanoparticle‐mediated scavenging of H_2_O_2_ is often more effective than a single‐component antioxidant, probably because of synergistic interactions and higher surface reactivity [122]. This efficient quenching of H_2_O_2_, together with the previously shown radical‐scavenging and lipid‐peroxidation inhibition, points to a multifunctional antioxidant potential of these NPs in mitigating oxidative stress in biological environments [123, 124].

##### 3.2.4.6 Superoxide Anion (O_2_•^−^) Scavenging Activity

**FIGURE 15:**
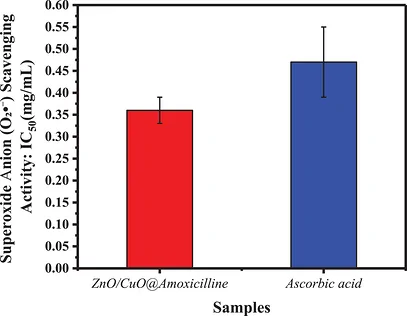
Superoxide anion (O_2_•^−^) scavenging activity of ZnO/CuO@Amoxicillin nanoparticles compared with ascorbic acid (IC_50_ values).

The ZnO/CuO@amoxicillin nanocomposite exhibited superoxide anion scavenging activity with an IC_50_ value of 0.36 ± 0.03 mg/mL. This value was lower than that of the ascorbic acid standard (0.47 ± 0.08 mg/mL, p < 0.05), indicating higher scavenging efficiency (Figure 15). The electron transfer to the superoxide anion is mechanistically facilitated by metal oxide surfaces, hence converting it into less reactive species such as hydrogen peroxide [118]. Moreover, surface defects and oxygen vacancies enhance this redox activity. Amoxicillin coating helps with the stabilization of reactive intermediates through hydrogen bonding and electron‐rich interactions, further amplifying radical quenching [125].The presence of Cu^2^^+^/Cu^+^ redox couples plays a central role in this process by enabling rapid electron exchange and catalytic cycling, which enhances superoxide neutralization efficiency [126].Additionally, oxygen vacancies and structural defects on ZnO and CuO surfaces act as active sites for adsorption and activation of superoxide radicals, further improving scavenging performance. The amoxicillin coating contributes by stabilizing reactive intermediates through hydrogen bonding and electron‐donating functional groups, thereby prolonging the antioxidant action at the nanoparticle interface [127]. These results suggest superior superoxide scavenging activities compared to previous literature on CuO, ZnO, or hybrid nanoparticles, due to synergistic redox interactions and increased surface reactivity [128]. This activity was combined with H_2_O_2_, FRAP, and lipid‐peroxidation inhibitory effects for comprehensive antioxidant potential and broad promise as multifunctional therapeutic agents in mitigating oxidative stress in cellular and tissue environments.

##### 3.2.4.7 Hydroxyl Radical (OH^•^) Scavenging Activity

**FIGURE 16:**
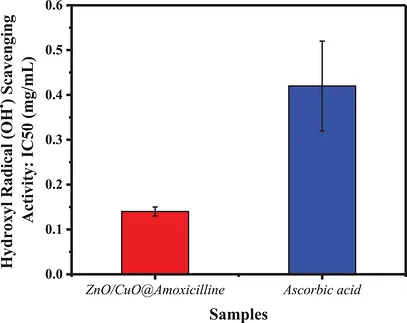
Hydroxyl radical (OH^•^) scavenging activity of ZnO/CuO@Amoxicillin nanoparticles compared with ascorbic acid (IC_50_ values).

The ZnO/CuO@amoxicillin nanocomposite exhibited hydroxyl radical scavenging activity with an IC_50_ value of 0.14 ± 0.01 mg/mL. This value was significantly lower than that of the ascorbic acid standard (0.42 ± 0.10 mg/mL, *p* < 0.01), indicating strong scavenging efficiency (Figure 16). This result underlines the high efficiency of the nanoparticles in neutralizing one of the most reactive and dangerous ROS in biological systems, capable of lipid peroxidation, DNA damage, and protein oxidation [114]. Mechanistically, the metal oxide cores (CuO and ZnO) favor electron transfer to the hydroxyl radicals, while structural features such as oxygen vacancies and high surface area enhance this quenching effect of the radicals [16]. This mechanism represents the final defensive barrier against oxidative stress, acting downstream of superoxide and hydrogen peroxide scavenging pathways. When integrated with superoxide dismutation, H_2_O_2_ decomposition, and electron‐transfer capacity (FRAP), this activity confirms a comprehensive, multi‐stage antioxidant defense system [129]. Further, in the case of coating with amoxicillin, electron‐rich sites and hydrogen bonding interactions stabilize transient radical intermediates [16]. These further amplify antioxidant efficacy. Comparisons with literature dealing with pure CuO, ZnO, or hybrid nanoparticles indicate that dual‐metal oxides realize better OH^•^ scavenging due to synergistic redox activity and optimized surface chemistry [116]. These results, together with H_2_O_2_, superoxide, FRAP, and TAC activities, underlined a multifunctional antioxidant capacity of the nanoparticles, turning them into promising candidates for mitigating oxidative stress in various biological applications.

##### 3.2.4.8 ABTS^•^^+^Scavenging Activity

**FIGURE 17:**
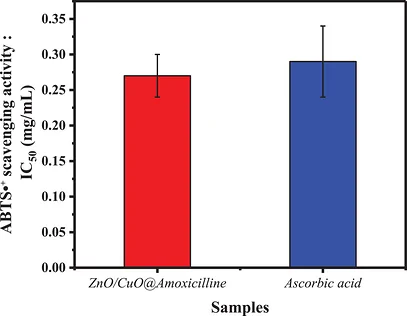
*ABTS^•^^+^* scavenging Activity of ZnO/CuO@Amoxicillin nanoparticles compared with ascorbic acid (IC_50_ values).

The ZnO/CuO@amoxicillin nanocomposite exhibited ABTS•^+^ radical scavenging activity with an IC_50_ value of 0.27 ± 0.03 mg/mL. This value was comparable to that of the ascorbic acid standard (0.29 ± 0.05 mg/mL, *p* > 0.05) (Figure 17). This assay is an indicative method of the antioxidant ability of the nanoparticles to quench both hydrophilic and lipophilic radical species and points toward a broad‐spectrum antioxidant activity [114]. Mechanistically, electron transfer of the redox‐active surfaces of CuO and ZnO therefore donates electrons to ABTS^+^ radicals, neutralizing them and breaking oxidative chain reactions [130]. The coexistence of Cu^2^^+^/Cu^+^ redox couples enhances this process by enabling rapid electron exchange and continuous catalytic activity at the nanoparticle interface. Additionally, oxygen vacancies and the high surface area of the nanocomposite improve radical adsorption and increase the accessibility of active sites, thereby boosting scavenging efficiency [131]. The amoxicillin coating provides polar functional groups and hydrogen‐bonding sites that stabilize transient radical intermediates. This stabilization prolongs the lifetime of antioxidant interactions and supports both ET and HAT pathways simultaneously [132]. The presence of oxygen vacancies and large surface areas increases the efficiency of electron transfer in these systems, whereas an amoxicillin coating further provides polar and hydrogen‐bonding sites for the stabilization of transient radical intermediates by prolonging antioxidant action [16]. Taken together, these compare well with previous reports on nanoparticles of single‐metal oxide systems, as hybrid CuO/ZnO analogs usually produce superior ABTS scavenging, likely due to the effective synergistic interaction between both the distinct metal oxides and optimization of the surface chemistry [118].

##### 3.2.4.9 DPPH Radical Scavenging Activity

**FIGURE 18:**
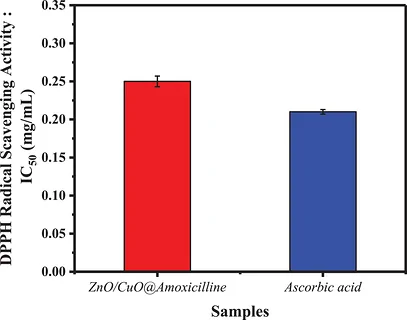
DPPH radical scavenging activity of ZnO/CuO@Amoxicillin nanoparticles compared with ascorbic acid (IC_50_ values).

The ZnO/CuO@amoxicillin nanocomposite exhibited DPPH radical scavenging activity with an IC_50_ value of 0.25 ± 0.07 mg/mL. This value was comparable to that of the ascorbic acid standard (0.21 ± 0.03 mg/mL, *p* > 0.05) (Figure 18). This reflects a high electron and/or hydrogen atom donation capacity with the effect of neutralizing the DPPH radicals and, consequently, the termination of oxidative chain reactions generally damaging lipids, proteins, and nucleic acids. Concretely, the inner CuO and ZnO cores introduce highly reactive surfaces which act as electron reservoirs, promoting a fast single‐electron transfer to the DPPH radical [70]. The presence of oxygen vacancies and surface defects further enhances such redox reactivity by giving additional active sites for radical interactions [133, 134]. Along these lines, the amoxicillin shell contributes polar functional groups capable of making hydrogen bonds to radical intermediates by stabilizing transient species and avoiding any propagation of radicals [135, 136]. Moreover, the presence of two metal oxide elements could be responsible for localized micro‐environments, significantly increasing the quenching efficiency due to a synergistic redox cycling between Cu^2^^+^/Cu^+^ and Zn^2^^+^ centers [116]. The Cu^2^^+^/Cu^+^ redox couple plays a critical role by facilitating continuous electron donation and regeneration of active sites, thereby sustaining the scavenging process [137]. Additionally, hydrogen atom transfer from surface hydroxyl groups or adsorbed species contributes to radical neutralization, complementing the electron transfer pathway. These defects increase the adsorption affinity of DPPH radicals onto the nanoparticle surface, improving reaction kinetics and overall efficiency [132].Thus, these hybrid CuO/ZnO nanoparticles exhibit superior or comparable DPPH‐scavenging efficiency compared to previously reported studies on PEG‐modified NiFe_2_O_4_ nanocomposites, ciprofloxacin‐loaded AgNPs, and CuO‐based nanotherapeutics [77, 138].

## 4 Conclusion

The present study demonstrates that the ZnO/CuO@amoxicillin nanocomposite is a promising multifunctional nanomaterial with diverse in vitro bioactivities. XRD, FTIR, and SEM analyses confirmed the formation of a crystalline biphasic metal oxide nanostructure with crystallite sizes of approximately 10–12 nm and a rough aggregated morphology. Although this morphology may favor surface interactions and drug incorporation, quantitative assessment of surface area, porosity, and drug‐loading characteristics requires further investigation.

The nanocomposite exhibited inhibitory effects against α‐amylase and α‐glucosidase, indicating potential relevance to the modulation of carbohydrate‐digesting enzymes. It also showed measurable anti‐inflammatory activity in the protein‐denaturation assay. In addition, the nanocomposite displayed antioxidant activity in both total antioxidant capacity and ferric reducing antioxidant power assays, together with scavenging activity toward superoxide anions, hydroxyl radicals, and hydrogen peroxide. Its ability to attenuate H_2_O_2_/FeCl_3_‐induced oxidative hemolysis in human erythrocytes further supports its protective activity against oxidative membrane damage under the tested in vitro conditions.

Collectively, the combination of structural characteristics and multiple in vitro bioactivities highlights the potential of ZnO/CuO@amoxicillin as a multifunctional bioactive nanomaterial. Nevertheless, the present findings should be interpreted within the limitations of the experimental design. In particular, the specific contribution of amoxicillin loading and potential synergistic effects between the metal oxide matrix and amoxicillin cannot be established without appropriate comparisons with free amoxicillin and the unloaded ZnO/CuO nanocomposite. Further studies should therefore focus on drug‐loading and release behavior, surface‐area and stability characterization, mechanistic investigations, and in vivo validation to determine the biological relevance, safety, and therapeutic potential of this nanocomposite.

## Author Contributions

**Hocine Sadam Nesrat**: conceptualization. **Ibtissam Laib** and **Salah Eddine Laouini**: methodology. **Salah Eddine Laouini**: software. **Abdelatif Aouadi** and **Djamila Hamada Saoud**: validation. **Mahmood M. S. Abdullah**: formal analysis. **Hocine Sadam Nesrat** and **Ibtissam Laib**: investigation. **Halima Zidane** and **Hafidha Terea**: resources. **Dalila Saidane–Mosbahi**: data curation. **Hocine Sadam Nesrat**: writing – original draft preparation. **Salah Eddine Laouini, Abdelatif Aouadi, Tomasz Trzepieciński**, and **Abderrhmane Bouafia**: writing – review and editing. **Tomasz Trzepieciński**: visualization. **Salah Eddine Laouini**: supervision. **Hocine Sadam Nesrat**: project administration. **Mahmood M. S. Abdullah**: funding acquisition. All authors have read and agreed to the published version of the manuscript.

## Funding

The funding for the study has been received from Algerian Directorate General for Scientific Research and Technological Development‐DGRSDT and El‐Oued University.

## Ethics Statement

The authors have nothing to report.

## Conflicts of Interest

The authors declare no conflicts of interest.

## Research Involving Human and Animals Rights

The authors have nothing to report.

## Data Availability

All data generated or analyzed during this study are included in this published article.

## References

[1] A. Sirelkhatim , S. Mahmud , A. Seeni , et al., “Review on Zinc Oxide Nanoparticles: Antibacterial Activity and Toxicity Mechanism,” Nano–Micro Lett 7 (2015): 219–242. 10.1007/s40820-015-0040-x PMC6223899 30464967

[2] R. Rajamohan , C. J. Raorane , S. C. Kim , S. Ashokkumar , and Y. R. Lee , “Novel Microwave Synthesis of Copper Oxide Nanoparticles and Appraisal of the Antibacterial Application,” Micromachines 14 (2023): 456. 36838156 10.3390/mi14020456 PMC9960782

[3] G. S. El‐Sayyad , E. S. R. El‐Sayed , S. H. Rizk , et al., “An Eco‐Friendly and Cost‐Effective Approach for the Synthesis of a Novel GA@CuO‐ZnO Nanocomposite: Characterization, Antimicrobial and Anticancer Activities,” RSC Advances 15 (2025): 513–523. 39763627 10.1039/d4ra04312j PMC11701889

[4] A. M. Elseman , A. E. Shalan , M. M. Rashad , A. M. Hassan , N. M. Ibrahim , and A. M. Nassar , “Easily Attainable New Approach to Mass Yield Ferrocenyl Schiff Base and Different Metal Complexes of Ferrocenyl Schiff Base Through Convenient Ultrasonication‐solvothermal Method,” Journal of Physical Organic Chemistry 30 (2017): e3639.

[5] F. Moradi , A. Ghaedi , Z. Fooladfar , and A. Bazrgar , “Recent Advance on Nanoparticles or Nanomaterials With Anti‐Multidrug Resistant Bacteria and Anti‐Bacterial Biofilm Properties: A Systematic Review,” Heliyon 9 (2023): e22105, 10.1016/j.heliyon.2023.e22105. 38034786 PMC10685370

[6] S. E. Diab , N. A. Tayea , B. H. Elwakil , A. A. E. M. Gad , D. A. Ghareeb , and Z. A. Olama , “Novel Amoxicillin‐Loaded Sericin Biopolymeric Nanoparticles: Synthesis, Optimization, Antibacterial and Wound Healing Activities,” International Journal of Molecular Sciences 23 (2022): 11654. 36232955 10.3390/ijms231911654 PMC9570309

[7] N. Akhtara and M. K. Bharali , “Recurrent Amoxicillin Exposure Disrupts Colonic Homeostasis Through Oxidative Stress, DNA Repair Dysregulation, and Gut Dysbiosis‐Driven Inflammation,” Chemico‐Biological Interactions 427 (2026): 111939. 41605271 10.1016/j.cbi.2026.111939

[8] H. S. Nesrat , D. Saidane‐Mosbahi , S. E. Laouini , et al., “Amoxicillin‐Functionalized Copper Oxide Nanoparticles: Synthesis, Characterization, and Multifunctional Biological Efficacy in Combating Infections and Oxidative Damage,” Journal of Sol‐Gel Science and Technology 116 (2025): 2923–2944.

[9] K. Jalil , S. Ahmad , N. ul Islam , S. Muhammad , Q. Jalil , and A. Ali , “Excellent Antibacterial and Anti‐Inflammatory Efficacy of Amoxicillin by AgNPs and Their Conjugates Synthesized Using Micromeria Biflora Crude Flavonoid Extracts,” Heliyon 10 (2024). 10.1016/j.heliyon.2024.e36752 PMC11399619 39281441

[10] Y. Jee , J. Carlson , E. Rafai , et al., “Antimicrobial Resistance: A Threat to Global Health,” Lancet Infectious Diseases 18 (2018): 939–940.30152350

[11] K. Bush and G. A. Jacoby , “Updated Functional Classification of β‐Lactamases,” Antimicrobial Agents and Chemotherapy 54 (2010): 969–976. 19995920 10.1128/AAC.01009-09 PMC2825993

[12] A. M. Nassar , A. M. Hassan , and S. S. Alabd , “Antitumor and Antimicrobial Activities of Novel Palladacycles With Abnormal Aliphatic CH Activation of Schiff Base 2‐[(3‐phenylallylidene) amino] phenol,” Synthesis and Reactivity in Inorganic, Metal‐Organic, and Nano‐Metal Chemistry 45 (2015): 256–270.

[13] H. C. Flemming , E. D. van Hullebusch , T. R. Neu , et al., “The Biofilm Matrix: Multitasking in a Shared Space,” Nature Reviews Microbiology 21 (2023): 70–86. 36127518 10.1038/s41579-022-00791-0

[14] K. Lakshmi , V. Rajarajeswari , and R. R. Muthuchudarkodi , “Synthesis of Copper Oxide Nanoparticles, Characterization and Photocatalytic Applications,” International Journal of Advanced Scientific Research and Management 4 (2019): 18.

[15] G. Zhang , J. Liu , Y. Zhu , T. Shen , and D. quan Yang , “Enhanced Antibacterial Efficacies, Corrosion Resistance, and Cytocompatibility of ZnO/CuO Composite Coatings Through Designed Sputtering Orders,” Applied Surface Science 635 (2023): 157724.

[16] T. T. T. Nguyen , Y. N. N. Nguyen , X. T. Tran , T. T. T. Nguyen , and T. Van Tran , “Green Synthesis of CuO, ZnO and CuO/ZnO Nanoparticles Using *Annona glabra* Leaf Extract for Antioxidant, Antibacterial and Photocatalytic Activities,” Journal of Environmental Chemical Engineering 11 (2023): 111003.

[17] A. M. Nassar , H. Salah , N. Hashem , M. Khodari , and H. F. Assaf , “Electrochemical Sensor Based on CuO Nanoparticles Fabricated From Copper Wire Recycling‐loaded Carbon Paste Electrode for Excellent Detection of Theophylline in Pharmaceutical Formulations,” Electrocatalysis 13 (2022): 154–164.

[18] R. M. Giráldez‐Pérez , E. M. Grueso , A. Carbonero , et al., “Synergistic Antibacterial Effects of Amoxicillin and Gold Nanoparticles: A Therapeutic Option to Combat Antibiotic Resistance,” Antibiotics 12 (2023): 1275. 37627696 10.3390/antibiotics12081275 PMC10451730

[19] M. Chamundeeswari , J. Jeslin , and M. L. Verma , “Nanocarriers for Drug Delivery Applications,” Environmental Chemistry Letters 17 (2019): 849–865.

[20] P. Ebenezer , S. P. S. N. B. S. Kumara , S. W. M. A. I. Senevirathne , et al., “Advancements in Antimicrobial Surface Coatings Using Metal/Metal‐Oxide Nanoparticles, Antibiotics, and Phytochemicals,” Nanomaterials (2025): 15, 10.3390/nano15131023. 40648730 PMC12251284

[21] J. M. V. Makabenta , A. Nabawy , C. H. Li , S. Schmidt‐Malan , R. Patel , and V. M. Rotello , “Nanomaterial‐Based Therapeutics for Antibiotic‐Resistant Bacterial Infections,” Nature Reviews Microbiology 19 (2021): 23–36. 32814862 10.1038/s41579-020-0420-1 PMC8559572

[22] K. I. Nassar , S. S. Teixeira , and M. P. F. Graça , “Sol–Gel‐Synthesized Metal Oxide Nanostructures: Advancements and Prospects for Spintronic Applications—A Comprehensive Review,” Gels 11 (2025): 657. 40868788 10.3390/gels11080657 PMC12385869

[23] R. Sui and P. Charpentier , “Synthesis of Metal Oxide Nanostructures by Direct Sol–Gel Chemistry in Supercritical Fluids,” Chemical Reviews 112 (2012): 3057–3082. 22394213 10.1021/cr2000465

[24] S. Das and V. C. Srivastava , “An Overview of the Synthesis of CuO‐ZnO Nanocomposite for Environmental and Other Applications,” Nanotechnology Reviews 7 (2018): 267–282.

[25] I. A. Vagena , M. A. Gatou , G. Theocharous , et al., “Functionalized ZnO‐Based Nanocomposites for Diverse Biological Applications: Current Trends and Future Perspectives,” Nanomaterials 14 (2024): 397. 38470728 10.3390/nano14050397 PMC10933906

[26] R. Goyat , J. Singh , A. Umar , et al., “Synthesis and Characterization of Nanocomposite Based Polymeric Membrane (PES/PVP/GO‐TiO_2_) and Performance Evaluation for the Removal of Various Antibiotics (amoxicillin, azithromycin & ciprofloxacin) From Aqueous Solution,” Chemosphere 353 (2024): 141542. 38428535 10.1016/j.chemosphere.2024.141542

[27] K. M. Mahnoor , A. Kazmi , T. Sultana , et al., “A Mechanistic Overview on Green Assisted Formulation of Nanocomposites and Their Multifunctional Role in Biomedical Applications,” Heliyon 11 (2025): e41654. 39916856 10.1016/j.heliyon.2025.e41654 PMC11800088

[28] M. Yuan , Q. Li , Y. Gao , et al., “Tunable Structured Metal Oxides for Biocatalytic Therapeutics,” Advanced Functional Materials 33 (2023): 2304271.

[29] K. Skłodowski , S. J. Chmielewska‐Deptuła , E. Piktel , P. Wolak , T. Wollny , and R. Bucki , “Metallic Nanosystems in the Development of Antimicrobial Strategies With High Antimicrobial Activity and High Biocompatibility,” International Journal of Molecular Sciences 24 (2023): 2104. 36768426 10.3390/ijms24032104 PMC9917064

[30] M. O. Boughanem , N. Touka , S. Mokhtari , et al., “Elaboration and Characterization of ZnO/CuO Nano‐Composites Produced by Sol–Gel Route for Supercapacitor Application,” Journal of Sol–Gel Science and Technology 114 (2025): 1040–1050.

[31] A. M. Nassar , A. H. Alanazi , M. M. Alzaid , and S. M. N. Moustafa , “Harnessing Wasted Mushroom Peel Aqueous Extract for Mycogenic Synthesis of Zinc Oxide Nanoparticles for Solar Photocatalysis and Antimicrobial Applications,” Biomass Convers Biorefinery 15 (2025): 20991–21006.

[32] F. Ajalloueian , S. Asgari , P. R. Guerra , et al., “Amoxicillin‐Loaded Multilayer Pullulan‐Based Nanofibers Maintain Long‐Term Antibacterial Properties With Tunable Release Profile for Topical Skin Delivery Applications,” International Journal of Biological Macromolecules 215 (2022): 413–423. 35700845 10.1016/j.ijbiomac.2022.06.054

[33] A. M. Hassan , A. M. Nassar , N. M. Ibrahim , A. M. Elsaman , and M. M. Rashad , “An Easy Synthesis of Nanostructured Magnetite‐Loaded Functionalized Carbon Spheres and Cobalt Ferrite,” Journal of Coordination Chemistry 66 (2013): 4387–4398.

[34] W. Brand‐Williams , M. E. Cuvelier , and C. Berset , (1995), https://doi.org/10.1016/S0023‐6438(95)80008‐5.

[35] G. Zengin , A. Aktumsek , G. O. Guler , Y. S. Cakmak , and E. Yildiztugay , “Antioxidant Properties of Methanolic Extract and Fatty Acid Composition of *Centaurea urvillei* DC. subsp. hayekiana Wagenitz,” Records of Natural Products 5 (2011): 123–132.

[36] N. Huda‐Faujan , A. Noriham , A. S. Norrakiah , and A. S. Babji , “Antioxidant Activity of Plants Methanolic Extracts Containing Phenolic Compounds,” African Journal of Biotechnology 8 (2009): 484–489.

[37] A. A. Karagözler , B. Erdaǧ , Y. Ç. Emek , and D. A. Uygun , “Antioxidant Activity and Proline Content of Leaf Extracts From *Dorystoechas hastata* ,” Food Chemistry 111 (2008): 400–407. 26047442 10.1016/j.foodchem.2008.03.089

[38] R. Re , N. Pellegrini , A. Proteggente , A. Pannala , M. Yang , and C. Rice‐Evans , “Antioxidant Activity Applying an Improved ABTS Radical Cation Decolorization Assay'',” Free Radical Biology and Medicine 26 (1999): 1231–1237. 10381194 10.1016/s0891-5849(98)00315-3

[39] M. Bannour , B. Fellah , G. Rocchetti , et al., “Phenolic Profiling and Antioxidant Capacity of *Calligonum azel* Maire, a Tunisian Desert Plant,” Food Research International 101 (2017): 148–154. 28941677 10.1016/j.foodres.2017.08.069

[40] A. Dolci and M. Panteghini , “Harmonization of Automated Hemolysis Index Assessment and Use: Is it Possible?” Clinica Chimica Acta 432 (2014): 38–43. 10.1016/j.cca.2013.10.012 24513329

[41] M. Daves , G. L. Salvagno , R. Cemin , et al., (2012).

[42] R. J. Ruch , S. Cheng , and J. E. Klaunig , “Prevention of Cytotoxicity and Inhibition of Intercellular Communication by Antioxidant Catechins Isolated From Chinese Green Tea,” Carcinogenesis 10 (1989): 1003–1008. 2470525 10.1093/carcin/10.6.1003

[43] K. S. J. Abirami , Wilson in AIP Conf Proc (2020): 030561.

[44] D. Malagoli , A Full‐length Protocol to Test Hemolytic Activity of Palytoxin on Human Erythrocytes (2007).

[45] A. Alam , T. jawaid , and P. Alam , “In Vitro Antioxidant and Anti‐Inflammatory Activities of Green Cardamom Essential Oil and In Silico Molecular Docking of Its Major Bioactives,” Journal of Taibah University & Sciences 15 (2021): 757–768.

[46] Y. Khelef , A. Chouikh , A. Rebiai , et al., (2019).

[47] J. Iqbal , A. Andleeb , H. Ashraf , et al., “Potential Antimicrobial, Antidiabetic, Catalytic, Antioxidant and ROS/RNS Inhibitory Activities of Silybum Marianum Mediated Biosynthesized Copper Oxide Nanoparticles,” RSC Advances 12 (2022): 14069–14083. 35558860 10.1039/d2ra01929a PMC9094097

[48] S. Kallithraka , C. Garcia‐Viguera , P. Bridle , and J. Bakker , (1995), https://doi.org/10.1002/pca.2800060509.

[49] S. Faisal , H. J. Abdullah , S. A. Shah , et al., “Bio‐Catalytic Activity of Novel *Mentha arvensis* Intervened Biocompatible Magnesium Oxide Nanomaterials,” Catalysts 11 (2021): 780.

[50] J. Ponmani , S. Kanakarajan , R. Selvaraj , and A. Kamalanathan , “Antioxidant Properties of Green Synthesized Silver Nanoparticles From *Sargassum wightii* ,” Saudi Journal of Medicine & Pharmaceutical Sciences 6 (2020): 516–525.

[51] A. Sumanth , V. Mishra , M. S. Ramachandra Rao , and T. Dixit , “Interface Analysis of CuO/ZnO Heterojunction for Optoelectronic Applications: An Experimental and Simulation Study,” Applied Materials Sciences (2023): 220, 10.1002/pssa.202300256.

[52] P. Makuła , M. Pacia , and W. Macyk , “How To Correctly Determine the Band Gap Energy of Modified Semiconductor Photocatalysts Based on UV–Vis Spectra,” Journal of Physical Chemistry Letters 9 (2018): 6814–6817. 30990726 10.1021/acs.jpclett.8b02892

[53] A. M. Nassar , Z. A. Alrowaili , A. A. M. Ahmed , B. A. Cheba , and S. Akhtar , “Facile Synthesis of New Composite, Ag‐Nps‐Loaded Core/Shell CdO/Co3O4 NPs, Characterization and Excellent Performance in Antibacterial Activity,” Applied Nanoscience 11 (2021): 419–428.

[54] A. Jabeen , A. Khan , P. Ahmad , et al., “Biogenic Synthesis of Levofloxacin‐Loaded Copper Oxide Nanoparticles Using *Cymbopogon citratus*: A Green Approach for Effective Antibacterial Applications,” Heliyon 10 (2024): e27018, 10.1016/j.heliyon.2024.e27018. 38501012 PMC10945134

[55] Z. Alhalili , “Green Synthesis of Copper Oxide Nanoparticles CuO NPs From *Eucalyptus globoulus* Leaf Extract: Adsorption and Design of Experiments,” Arab Journal of Chemistry 15 (2022): 103739.

[56] A. M. Hassan , A. M. Nassar , Y. Z. Hussien , and A. N. Elkmash , “Synthesis, Characterization and Biological Evaluation of Fe (III), Co (II), Ni (II), Cu (II), and Zn (II) Complexes With Tetradentate Schiff Base Ligand Derived From Protocatechualdehyde With 2‐Aminophenol,” Applied Biochemistry & Biotechnology 167 (2012): 581–594. 22576963 10.1007/s12010-012-9709-5

[57] H. Terea , A. Rebiai , D. Selloum , and M. L. Tedjani , “Cellulose/ZnO Nanoparticles (CNC/ZnO NPs): Synthesis, Characterization, and Evaluation of Their Antibacterial and Antifungal Activities,” Cellulose 31 (2024): 5027–5042.

[58] H. Terea , D. Selloum , A. Rebiai , A. Bouafia , and O. B. Mya , “Preparation and Characterization of Cellulose/ZnO Nanoparticles Extracted From Peanut Shells: Effects on Antibacterial and Antifungal Activities,” Biomass Convers Biorefinery 14 (2024): 19489–19500.

[59] M. Roudgar‐Amoli , A. Alizadeh , E. Abedini , and Z. Shariatinia , “Delafossite CuCoO_2_/ZnO Derived From ZIF‐8 Heterojunctions as Efficient Photoelectrodes for Dye‐sensitized Solar Cells,” RSC Advances 13 (2023): 14825–14840. 37197189 10.1039/d3ra01595e PMC10184138

[60] A. Ashour , E. E. Assem , and E. R. Shaaban , “Investigation of Dilute Aluminum Doped Zinc Oxide Thin Films: Structural and Morphological Properties for Varies Applications,” Journal of Ovonic Research 18 (2022): 699–711.

[61] D. R. A. Preethi and A. Philominal , “Green Synthesis of Pure and Silver Doped Copper Oxide Nanoparticles Using *Moringa oleifera* Leaf Extract,” Mater Lett X 13 (2022): 100122.

[62] S. I. Yusuf , S. J. Mohammad , and M. H. Ali , “Scherrer and Williamson‐Hall Estimated Particle Size Using XRD Analysis for Cast Aluminum Alloys,” Journal of Theoretical Applied Physics 18 (2024): si‐AICIS23.17, 10.57647/j.jtap.2024.si-AICIS23.17.

[63] D. C. Deka , V. Kumar , C. Prasad , et al., “ *Oroxylum indicum*–A Medicinal Plant of North East India: An Overview of Its Nutritional, remedial, and prophylactic properties,” Journal of Applied Pharmaceutical Sciences 3 (2013): S104–S112.

[64] K. R. B. Singh , V. Nayak , J. Singh , A. K. Singh , and R. P. Singh , “Potentialities of Bioinspired Metal and Metal Oxide Nanoparticles in Biomedical Sciences,” RSC Advances 11 (2021): 24722–24746. 35481029 10.1039/d1ra04273d PMC9036962

[65] S. Dasgupta , S. Tarafder , A. Bandyopadhyay , and S. Bose , “Effect of Grain Size on Mechanical, Surface and Biological Properties of Microwave Sintered Hydroxyapatite,” Material Science & Engineering C 33 (2013): 2846–2854. 10.1016/j.msec.2013.03.004 23623105

[66] G. A. Govindasamy , R. B. S. M. N. Mydin , S. Sreekantan , and N. H. Harun , “Compositions and Antimicrobial Properties of Binary ZnO–CuO Nanocomposites Encapsulated Calcium and Carbon From *Calotropis gigantea* Targeted for Skin Pathogens,” Science Reports 11 (2021): 99. 10.1038/s41598-020-79547-w PMC7794424 33420110

[67] J. O. Adeyemi , D. C. Onwudiwe , and A. O. Oyedeji , “Biogenic Synthesis of CuO, ZnO, and CuO–ZnO Nanoparticles Using Leaf Extracts of *Dovyalis caffra* and Their Biological Properties,” Molecules 27 (2022): 3206. 35630680 10.3390/molecules27103206 PMC9144262

[68] J. P. Selvam and P. Ponnusamy , “Green Synthesis of CuO and ZnO Nanoparticles From Edible Mushrooms: Characterization and Evaluation of Antioxidant, Antimicrobial, Anti‐Inflammatory and Cytotoxicity Activities,” Chemical Physics Impact 12 (2026): 100985.

[69] S.‐H. Cha , J. Hong , M. McGuffie , B. Yeom , J. S. VanEpps , and N. A. Kotov , “Shape‐Dependent Biomimetic Inhibition of Enzyme by Nanoparticles and Their Antibacterial Activity,” ACS Nano 9 (2015): 9097–9105. 26325486 10.1021/acsnano.5b03247

[70] H. K. Abid , A. B. Siddique , A. Abbas , et al., “Biogenic Synthesis of ZnO NPs, CuO NPs, and ZnO/CuO Nanocomposites for Facile Degradation of Organic Pollutants and Biomedical Applications,” Nanoscale Advances 7 (2025): 6145–6157. 40880602 10.1039/d5na00583c PMC12372208

[71] M. A. Amin , N. A. Algamdi , M. S. Waznah , et al., “An Insight Into Antimicrobial, Antioxidant, Anticancer, and Antidiabetic Activities of Trimetallic Se/ZnO/CuO Nanoalloys Fabricated by Aqueous Extract of *Nitraria retusa* ,” Journal of Cluster Science 36 (2025): 19.

[72] N. R. Shailaja , M. Arulmozhi , B. Balraj , and C. Siva , “ *Corallocarpus epigaeus* Mediated Synthesis of ZnO/CuO Integrated ZrO_2_ Nanoparticles for Enhanced in‐vitro Antibacterial, Antifungal and Antidiabetic Activities,” Journal of the Indian Chemical Society 100 (2023): 100991.

[73] K. Neethidevan , K. Ravichandran , M. Ayyanar , et al., “ *Wattakaka volubilis* Powered Green Synthesized CuO, NiO and ZnO Nanoparticles for Cost‐Effective Biomedical Applications,” Biomass Convers Biorefinery 14 (2024): 31575–31589.

[74] A. F. El‐Sayed , W. M. Aboulthana , M. A. Sherief , G. T. El‐Bassyouni , and S. M. Mousa , “Synthesis, Structural, Molecular Docking, and In Vitro Biological Activities of Cu‐doped ZnO Nanomaterials,” Scientific Reports 14 (2024): 9027. 38641640 10.1038/s41598-024-59088-2 PMC11031592

[75] R. Papitha , V. Hadkar , N. K. Sishu , S. Arunagiri , S. M. Roopan , and C. I. Selvaraj , “Green Synthesis of CuO/TiO2 and ZnO/TiO2 Nanocomposites Using *Parkia timoriana* Bark Extract: Enhanced Antioxidant and Antidiabetic Activities for Biomedical Applications,” Ceramics International 50 (2024): 39109–39121.

[76] X. Liu , M. Wu , C. Li , et al., “Interaction Structure and Affinity of Zwitterionic Amino Acids With Important Metal Cations (Cd2+, Cu2+, Fe3+, Hg2+, Mn2+, Ni2+ and Zn2+) in Aqueous Solution: A Theoretical Study,” Molecules 27 (2022): 2407. 35458605 10.3390/molecules27082407 PMC9028192

[77] I. Laib , H. A. Mohammed , S. E. Laouini , et al., “Cutting‐Edge Nano‐Therapeutics: Silver Nanoparticles Loaded With Ciprofloxacin for Powerful Antidiabetic, Antioxidant, Anti‐inflammatory, and Antibiotic Action Against Resistant Pathogenic Bacteria,” International Journal of Food Science and Technology 60 (2025): vvaf024.

[78] H. A. Rudayni , M. H. Shemy , M. Aladwani , et al., “Synthesis and Biological Activity Evaluations of Green ZnO‐Decorated Acid‐Activated Bentonite‐Mediated Curcumin Extract (ZnO@ CU/BE) as Antioxidant and Antidiabetic Agents,” Journal of Functional Biomaterials 14 (2023): 198. 37103288 10.3390/jfb14040198 PMC10146122

[79] M. Pandey , M. Singh , K. Wasnik , et al., “Targeted and Enhanced Antimicrobial Inhibition of Mesoporous ZnO–Ag_2_O/Ag, ZnO–CuO, and ZnO–SnO_2_ composite Nanoparticles,” ACS omega 6 (2021): 31615–31631. 34869986 10.1021/acsomega.1c04139 PMC8637601

[80] V. Karthick , A. A. Zahir , M. Ayyanar , et al., “Optimization and Characterization of Eco‐Friendly Formulated ZnO NPs in Various Parameters: Assessment of Its Antidiabetic, Antioxidant and Antibacterial Properties,” Biomass Convers Biorefinery 14 (2024): 24567–24581.

[81] H. Hosseinzadeh , H. Oveisi , and A. Meshkini , “Functionalized ZnFe2O4@ Mesoporous Silica Nano‐support for Lipase Enzyme Immobilization: Enhanced Biocatalysis and Antibacterial Activity for Food Industry Applications,” Food Bioscience 61 (2024): 104985.

[82] A. Bouafia , A. Boutalbi , S. E. Laouini , et al., “Copper Oxide Nanoparticles: Characterization, Photocatalysis, and Biomedical Applications,” Chemical Engineering & Technology (2025): 48, 10.1002/ceat.70073.

[83] A. H. Said , F. Shaibah , M. Moustafa , and R. B. Elamary , “Plant‐Mediated Nickel Oxide Nanoparticles Show Species‐Dependent Antibacterial, Antioxidant, Anti‐inflammatory and Antidiabetic Activities,” Scientific Reports 15 (2025): 31096. 40851031 10.1038/s41598-025-15951-4 PMC12375740

[84] H. Agarwal and V. K. Shanmugam , “A Review on Anti‐inflammatory Activity of Green Synthesized Zinc Oxide Nanoparticle: Mechanism‐Based Approach,” Bioorganic Chemistry 94 (2020): 103423. 31776035 10.1016/j.bioorg.2019.103423

[85] A. M. H. Al‐Rajhi , R. Yahya , M. M. Bakri , R. Yahya , and T. M. Abdelghany , “In Situ Green Synthesis of Cu‐doped ZnO Based Polymers Nanocomposite With Studying Antimicrobial, Antioxidant and Anti‐Inflammatory Activities,” Applied Biology & Chemistry 65 (2022): 35.

[86] Q. Gao , S. Liu , Z. Guo , D. Baimanov , M. Chen , and L. Wang , “Nanozyme‐Biological Interfaces: A Review on Mechanisms and Applications From Natural Biomolecular Corona to Artificial Surface Modifications,” ACS Applied Nano Materials 8 (2025): 22070–22085.

[87] H. D. Huynh , T. H. T. Thi , T. X. T. Thi , et al., “Recent Insights Into Protein‐Polyphenol Complexes: Molecular Mechanisms, Processing Technologies, Synergistic Bioactivities, and Food Applications,” Molecules 31 (2026): 287. 41599337 10.3390/molecules31020287 PMC12844103

[88] G. Sabeena , S. Rajaduraipandian , T. Manju , et al., “In Vitro Antidiabetic and Anti‐Inflammatory Effects of Fe‐Doped CuO‐rice Husk Silica (Fe‐CuO‐SiO2) Nanocomposites and Their Enhanced Innate Immunity in Zebrafish,” Journal of King Saud University‐Computer and Information Sciences 34 (2022): 102121.

[89] S. Faisal , H. Jan , I. A. Abdullah , et al., “In Vivo Analgesic, Anti‐Inflammatory, and Anti‐Diabetic Screening of Bacopa Monnieri‐Synthesized Copper Oxide Nanoparticles,” ACS Omega 7 (2022): 4071–4082. 35155901 10.1021/acsomega.1c05410 PMC8829860

[90] G. Fontana , M. Licciardi , S. Mansueto , D. Schillaci , and G. Giammona , “Amoxicillin‐Loaded Polyethylcyanoacrylate Nanoparticles: Influence of PEG Coating on the Particle Size, Drug Release Rate and Phagocytic Uptake,” Biomaterials 22 (2001): 2857–2865. 11561891 10.1016/s0142-9612(01)00030-8

[91] D. Barani , A. Bouafia , S. E. Laouini , et al., “Enhanced Antioxidant, Anti‐Inflammatory, and Photocatalytic Properties of α‐Tocopherol‐Coated Copper Oxide Nanoparticles: Synthesis, Characterization, and Multifunctional Applications,” International Journal of Food Science and Technology (2025), 10.1093/ijfood/vvaf051.

[92] I. Laib , A. Bouafia , S. E. Laouini , et al., “Ciprofloxacin‐Loaded Copper Oxide Nanoparticles: Cutting‐Edge Multifunctional Nano‐Therapeutics With Superior Antidiabetic, Antioxidant, Anti‐Inflammatory, and Antibacterial Potency Against Drug‐Resistant Pathogens,” Journal of Crystal Growth 653 (2025): 128074.

[93] J. Bao , T. Huang , Z. Wang , et al., “3D Graphene/Copper Oxide Nano‐Flowers Based Acetylcholinesterase Biosensor for Sensitive Detection of Organophosphate Pesticides,” Sensors Actuators B Chem 279 (2019): 95–101.

[94] A. A. Almehizia , A. M. Naglah , S. S. Aljafen , A. S. Hassan , and W. M. Aboulthana , “Assessment of the In Vitro Biological Activities of Schiff Base‐Synthesized Copper Oxide Nanoparticles as an Anti‐Diabetic, Anti‐Alzheimer, and Anti‐Cancer Agent,” Pharmaceutics 17 (2025): 180. 40006547 10.3390/pharmaceutics17020180 PMC11859031

[95] E. Yadav and P. Yadav , “Biofabricated Zinc Oxide Nanoparticles Impair Cognitive Function via Modulating Oxidative Stress and Acetylcholinesterase Level in Mice,” Environmental Toxicology 36 (2021): 572–585. 33247493 10.1002/tox.23062

[96] H. Kotrange , A. Najda , A. Bains , R. Gruszecki , P. Chawla , and M. M. Tosif , “Metal and Metal Oxide Nanoparticle as a Novel Antibiotic Carrier for the Direct Delivery of Antibiotics,” International Journal of Molecular Sciences 22 (2021): 9596. 34502504 10.3390/ijms22179596 PMC8431128

[97] D. A. Demirezen , Y. Ş. Yıldız , and D. D. Yılmaz , “Amoxicillin Degradation Using Green Synthesized Iron Oxide Nanoparticles: Kinetics and Mechanism Analysis'' *Environ* ,” Nanotechnology Monitoring Management 11 (2019): 100219.

[98] J. K. Suthar , A. Vaidya , and S. Ravindran , “Size, Surface Properties, and Ion Release of Zinc Oxide Nanoparticles: Effects on Cytotoxicity, Dopaminergic Gene Expression, and Acetylcholinesterase Inhibition in Neuronal PC‐12 Cells,” Biological Trace Element Research 202 (2024): 2254–2271. 37713055 10.1007/s12011-023-03832-8

[99] N. Baskaran and A. Subash , “Green Synthesis and Characterization of Zinc Oxide Nanoparticles (ZnO NPs) From *Camellia sinensis* Leaf Extract and Its Potential of Antibacterial Activity and Acetyl Cholinesterase Inhibitory Activities,” Journal of PharmaceuticalResearch International 33 (2021): 134–147.

[100] S. Mouna , S. Hajji , and H. Tounsi , “Waste to Health: Green Synthesis of Zn Loaded LTA Zeolite Prepared From Waste Glass and Aluminum Scrap With High Antioxidant and Antimicrobial Activities,” J Clean Prod 434 (2024): 139946.

[101] A. H. Jawad , N. S. A. Mubarak , and A. S. Abdulhameed , “Hybrid Crosslinked Chitosan‐Epichlorohydrin/TiO2 Nanocomposite for Reactive Red 120 Dye Adsorption: Kinetic, Isotherm, Thermodynamic, and Mechanism Study,” Journal of Polymers and the Environment 28 (2020): 624–637.

[102] H. Naeem , M. Ajmal , R. B. Qureshi , S. T. Muntha , M. Farooq , and M. Siddiq , “Facile Synthesis of Graphene Oxide–Silver Nanocomposite for Decontamination of Water From Multiple Pollutants by Adsorption,” Journal of Environmental Management 230 (2019): 199–211. 30286349 10.1016/j.jenvman.2018.09.061

[103] R. S. Shinde , R. A. More , V. A. Adole , et al., “Design, Fabrication, Antitubercular, Antibacterial, Antifungal and Antioxidant Study of Silver Doped ZnO and CuO Nano Candidates: A Comparative Pharmacological Study,” Current Research in Green and Sustainable Chemistry 4 (2021): 100138.

[104] M. S. Yalçın , S. Özdemir , V. Prokopiuk , et al., “Toxicity, Antibacterial, Antioxidant, Antidiabetic, and DNA Cleavage Effects of Dextran‐Graft‐Polyacrylamide/Zinc Oxide Nanosystems,” Current Microbiology 81 (2024): 437. 39487865 10.1007/s00284-024-03953-w

[105] T. F. Khan , M. Muhyuddin , S. Irum , M. A. Ali , S. W. Husain , and M. A. Basit , “Comparing the Antioxidant and Hemolytic Activity of Wet‐Chemically Synthesized ZnO, ZnS, and ZnO/ZnS Nanocomposite,” Inorganic Chemistry Communications 174 (2025): 113902.

[106] A. M. Ali , A. M. Hamed , M. A. Taher , et al., “Fabrication of Antibacterial and Antioxidant ZnO‐Impregnated Amine‐Functionalized Chitosan Bio‐Nanocomposite Membrane for Advanced Biomedical Applications,” Molecules 28 (2023): 7034. 37894513 10.3390/molecules28207034 PMC10608820

[107] E. Lizundia , I. Armentano , F. Luzi , et al., “Synergic Effect of Nanolignin and Metal Oxide Nanoparticles Into Poly (l‐lactide) Bionanocomposites: Material Properties, Antioxidant Activity, and Antibacterial Performance,” ACS Applied Bio Materials 3 (2020): 5263–5274. 10.1021/acsabm.0c00637 35021701

[108] G. S. Thirumoorthy , O. Balasubramaniam , P. Kumaresan , P. Muthusamy , and K. Subramani , “ *Tetraselmis indica* Mediated Green Synthesis of Zinc Oxide (ZnO) Nanoparticles and Evaluating Its Antibacterial, Antioxidant, and Hemolytic Activity,” Bionanoscience 11 (2021): 172–181.

[109] D. Doudi , N. Mahboub , N. Gheraissa , et al., “Eco‐Friendly Synthesis and Characterization of ZnO and Mg‐Ag‐Doped ZnO Nanoparticles Using *Phoenix dactylifera* L. seeds: Exploring Biological Activity and Structural Properties,” Biomass Convers Biorefinery 14 (2024): 1–21.

[110] S. O. Mohammed , M. M. H. Khalil , I. M. El‐Sewify , and A. Radwan , “Nd‐Doped CuO/ZnO and ZnO/CuO Heterojunctions for Simultaneous UV Blocking and Malachite Green Detoxification,” Scientific Reports 15 (2025): 34063. 41028124 10.1038/s41598-025-13945-w PMC12484677

[111] C. Wang , L. Yue , S. Wang , et al., “Role of Electric Field and Reactive Oxygen Species in Enhancing Antibacterial Activity: A Case Study of 3D Cu Foam Electrode With Branched CuO–ZnO NWs,” Journal of Physical Chemistry C 122 (2018): 26454–26463.

[112] M. Aggarwal , P. Ghosh , K. P. Mohithraj , et al., “A Hydrothermally Transformed Multifunctional Proteinaceous Nanoparticle Biosealant With Intrinsic Light‐responsive Redox Cycling for Self‐Sterilizing and Accelerated Wound Healing,” Journal of Materials Chemistry B 14 (2026): 2028–2040. 41603295 10.1039/d5tb02363g

[113] Y. Zhou , Y. Zhang , Z. Li , et al., “Oxygen Reduction Reaction Electrocatalysis Inducing Fenton‐Like Processes With Enhanced Electrocatalytic Performance Based on Mesoporous ZnO/CuO Cathodes: Treatment of Organic Wastewater and Catalytic Principle,” Chemosphere 259 (2020): 127463. 32599388 10.1016/j.chemosphere.2020.127463

[114] M. Jeevarathinam and I. V. Asharani , “Synthesis of CuO, ZnO Nanoparticles, and CuO‐ZnO Nanocomposite for Enhanced Photocatalytic Degradation of Rhodamine B: A Comparative Study,” Scientific Reports 14 (2024): 9718. 38678108 10.1038/s41598-024-60008-7 PMC11577056

[115] A. Tabet , A. Bouafia , K. Al‐Essa , et al., “Unveiling the Synthesis, Characterization, and Multifunctional Applications of Ca5(PO4)3F@Ca5P8 Nanocomposites From Phosphate Waste for Antioxidant Activity and Evans Bleu Decomposition,” ChemistrySelect 10 (2025): e202406193, 10.1002/slct.202406193.

[116] J. O. Adeyemi , D. C. Onwudiwe , and A. O. Oyedeji , “Biogenic Synthesis of CuO, ZnO, and CuO–ZnO Nanoparticles Using Leaf Extracts of *Dovyalis caffra* and Their Biological Properties,” Molecules 27 (2022): 3206. 35630680 10.3390/molecules27103206 PMC9144262

[117] I. F. F. Benzie and M. Devaki , “The Ferric Reducing/Antioxidant Power (FRAP) Assay for Non‐Enzymatic Antioxidant Capacity: Concepts, Procedures, Limitations and Applications,” Measurement of Antioxidant Activity and Capacity: Recent Trends and Applications 1 (2018): 77–106.

[118] K. Madeshwaran and R. Venkatachalam , “Green Synthesis of Bimetallic ZnO–CuO Nanoparticles Using *Annona muricata* L. extract: Investigation of antimicrobial, antioxidant, and anticancer properties,” Journal of Industrial and Engineering Chemistry 140 (2024): 454–467.

[119] T. A. Orshiso , E. A. Zereffa , H. C. A. Murthy , et al., “Biosynthesis of *Artemisia abyssinica* Leaf Extract‐Mediated Bimetallic ZnO–CuO Nanoparticles: Antioxidant, Anticancer, and Molecular Docking Studies,” ACS omega 8 (2023): 41039–41053. 37969984 10.1021/acsomega.3c01814 PMC10633890

[120] N. Iravani , M. Mohammadi , and Z. Hossaini , “Green Synthesis of New Spiropyrrole Derivatives by Employing Activated Carbonyl Compounds and Graphen Oxide Decorated on Ag@ CuO/ZnO NPs or CuO/ZnO NPs: Theoretical and Biological Study,” Applied Organometallic Chemistry 39 (2025): e7859.

[121] J. Li , J. Zeng , L. Jia , and W. Fang , “Investigations on the Effect of Cu2+/Cu1+ Redox Couples and Oxygen Vacancies on Photocatalytic Activity of Treated LaNi1− xCuxO3 (x = 0.1, 0.4, 0.5),” International Journal of Hydrogen Energy 35 (2010): 12733–12740.

[122] S. Ihsan , H. Munir , Z. Meng , et al., “Tragacanth Gum‐Based Copper Oxide Nanoparticles: Comprehensive Characterization, Antibiofilm, Antimicrobial and Photocatalytic Potentials,” International Journal of Biological Macromolecules 268 (2024): 131600. 38631575 10.1016/j.ijbiomac.2024.131600

[123] H. T. Gebrie , M. A. Assege , D. S. Meshesha , et al., “Biosynthesis and Characterization of Copper Oxide Nanoparticles From *Plumbago zeylanica* Leaf Extract for Antibacterial and Antioxidant Activities,” Scientific Reports 15 (2025): 30656. 40835861 10.1038/s41598-025-10700-z PMC12368077

[124] G. Govindasamy and A. B. Ponnusami , “Development and Evaluation of Hydrogen Peroxide Mediated Zinc Oxide Photocatalytic Nanoparticles From Peepal (*Ficus religiosa*) Leaf Extract for the Treatment of Actual Tannery Wastewater,” Environ Science & Water Research Technology 11 (2025): 508–523.

[125] R. A. M. Yahya , A. E. Azab , and K. E. M. Shkal , “Effects of Copper Oxide and/or Zinc Oxide Nanoparticles on Oxidative Damage and Antioxidant Defense System in Male Albino Rats,” EAS Journal of Pharmacy & Pharmacology 1 (2019): 135–144.

[126] C. Ma , J. Wu , B. Ji , B. Li , X. Li , and C. Wang , “Cu+/Cu2+ Mixed‐Valence Synergistic Catalytic Sites in BiVO_4_ Enhance Charge Transfer and Efficient Photodegradation of Ofloxacin,” Applied Surface Science 646 (2025): 164086.

[127] A. Squarcina , A. Santoro , N. Hickey , et al., “Neutralization of Reactive Oxygen Species at Dinuclear Cu (II)‐Cores: Tuning the Antioxidant Manifold in Water by Ligand Design,” ACS Catalysis 10 (2020): 7295–7306.

[128] J. Daimari and A. K. Deka , “Anticancer, Antimicrobial And Antioxidant Activity of CuO–ZnO Bimetallic Nanoparticles: Green Synthesised From *Eryngium foetidum* Leaf Extract,” Scientific Reports 14 (2024): 19506. 39174638 10.1038/s41598-024-69847-w PMC11341821

[129] S. Ngwenya , N. J. Sithole , K. Ramachela , et al., “Eco‐Friendly Synthesis of ZnO, CuO, and ZnO/CuO Nanoparticles Using Extract of Spent *Pleurotus ostreatus* Substrate, and Their Antioxidant and Anticancer Activities,” Discover Nano 20 (2025): 35. 39945970 10.1186/s11671-025-04199-6 PMC11825426

[130] S. Ghaffar , A. Abbas , M. Naeem‐ul‐Hassan , et al., “Improved Photocatalytic and Antioxidant Activity of Olive Fruit Extract‐Mediated ZnO Nanoparticles,” Antioxidants 12 (2023): 1201. 37371931 10.3390/antiox12061201 PMC10295640

[131] K. S. Ranjith , C.‐L. Dong , Y.‐R. Lu , et al., “Evolution of Visible Photocatalytic Properties of Cu‐Doped CeO2 Nanoparticles: Role of Cu2+‐Mediated Oxygen Vacancies and the Mixed‐Valence States of Ce Ions,” ACS Sustainable Chemistry & Engineering 6 (2018): 8536–8546.

[132] A. U. Khan , Q. U. Khan , K. Tahir , et al., “A *Tagetes minuta* Based Eco‐Benign Synthesis of Multifunctional Au/MgO Nanocomposite With Enhanced Photocatalytic, Antibacterial and DPPH Scavenging Activities,” Materials & Science Engineering C 126 (2021): 112146. 10.1016/j.msec.2021.112146 34082957

[133] W. Azabi , N. Gherraf , A. Romero , and J. A. A. Abdullah , “Synergetic Green Synthesis of CuO, ZnO, and CuO‐ZnO Nanocomposite Nanoparticles Using *Genista hispanica* L. extract for Enhanced Photocatalytic and Antioxidant Properties,” Research on Chemical Intermediates 51 (2025): 1–27.

[134] S. E. Laouini , A. Bouafia , M. Azzi , et al., “Synthesis and Characterization of Amoxicillin‐Functionalized Ag/AgCl Nanoparticles: A Promising Multifunctional Platform for Next‐Generation Nanomedicine,” Journal of Biomedical Materials Research Part B Applied Biomaterials 113 (2025): e35661. 41017736 10.1002/jbm.b.35661

[135] R. Kumar , K. Kumar , S. Sharma , N. Thakur , and N. Thakur , “Multifunctional Properties of Microwave Assisted CuO/Cu2O‐ZnO Mixed Metal Oxide Nanocomposites,” Journal of Materials Science: Materials in Electronics 34 (2023): 1255.

[136] I. Laib , N. Gheraissa , A. Benaissa , et al., “Tailoring Innovative Silver Nanoparticles for Modern Medicine: The Importance of Size and Shape Control and Functional Modifications,” Materials Today Bio 21 (2025): 102071. 10.1016/j.mtbio.2025.102071 PMC12302930 40727080

[137] L. Cui , J. Wu , J. Li , Y. Ge , and H. Ju , “Electrochemical Detection of Cu2+ Through Ag Nanoparticle Assembly Regulated by Copper‐Catalyzed Oxidation of Cysteamine,” Biosensors & Bioelectronics 55 (2014): 272–277. 24389390 10.1016/j.bios.2013.11.081

[138] H. A. Mohammed , A. Bouafia , S. E. Laouini , et al., “PEG‐Modified NiFe_2_O_4_ Nanocomposites: Synthesis, Characterization, and Multifunctional Biomedical Applications in Antioxidant, Anti‐Inflammatory, Photoprotective, Antiemolytic, and Antibacterial Therapies,” Applied Organometallic Chemistry 39 (2025): e70212.

